# A nomological network for misophonia in two German samples using the S-Five model for misophonia

**DOI:** 10.3389/fpsyg.2022.902807

**Published:** 2022-12-21

**Authors:** Nico Remmert, Antonia Jebens, Rebecca Gruzman, Jane Gregory, Silia Vitoratou

**Affiliations:** ^1^Department of Education and Psychology, Freie Universität Berlin, Berlin, Germany; ^2^Department of Psychometrics and Measurement Lab, Biostatistics and Health Informatics King’s College London, Institute of Psychiatry, Psychology and Neurosciences, London, United Kingdom; ^3^Department of Psychology, Medical School Berlin, Berlin, Germany; ^4^Department of Experimental Psychology, University of Oxford, Oxford, United Kingdom

**Keywords:** misophonia, nomological network, psychometrics, selective sound sensitivity syndrome scale S-Five, construct validation

## Abstract

The Selective Sound Sensitivity Syndrome Scale (S-Five) is a contemporary and multidimensional self-report instrument measuring different aspects of misophonia. The five-factor scale consists of 25 items measuring the severity of the misophonic experience. The items capture misophonia in relation to internalising and externalising appraisals, perceived threat, aggressive behavior (outbursts), and adverse impact on individuals’ lives. It is complemented by a trigger checklist (S-Five-T), measuring the emotional nature and intensity of reactions to sensory triggers. In this work, we administered the S-Five in two German samples with a majority of individuals with significant misophonia. The S-Five and the supplementary S-Five-T were both translated into German using a rigorous translation procedure (i.e., TRAPD) and were separately tested in large German community samples. Psychometric analyses included the evaluation of the factor structure, measurement invariance with respect to age and gender, reliability (internal consistency and stability over time), and an extensive examination of the construct validity in a proposed nomological network. The nomological network we explore in this work consists of several constructs including different misophonic manifestations, anger and aggression, disgust propensity, anxiety sensitivity, depression, obsessive–compulsive traits, and functional impairment in different life domains. Results indicate evidence in line with the nomological network as demonstrated by strong correlations between the S-Five dimensions and convergent measures. All S-Five dimensions strongly correlated with overall misophonic symptoms (*r* ≥ 0.53). Internalising appraisals were highly associated with insight into excessive or disproportionate reactions to sounds (*r* ≥ 0.59), externalising appraisals with anger and irritability (*r* ≥ 0.46), threat with trait anxiety and dysregulation facets (*r* ≥ 0.62), aggressive behavior (outbursts) with anger and behavioral dysregulation (*r* ≥ 0.70), and impact with distress and functional impairment (*r* ≥ 0.64). The results demonstrate that the S-Five has a robust five-factor structure and allows to draw reliable and valid conclusions about misophonic experiences in German samples. The proposed nomological network gives an initial insight into the nature of misophonia and provides a formalized fundament to develop and test further hypotheses about misophonia in a more sophisticated and symptom-oriented way.

## Introduction

Misophonia is a disorder related to decreased tolerance to certain sounds (Swedo et al., 2022), most commonly sounds related to eating, nose and throat sounds, and repetitive environmental sounds (Vitoratou et al., 2021a). Individuals with misophonia can experience profound distress and functional impairment from their emotional, physical and behavioral responses to these sounds (e.g., Jastreboff and Jastreboff, 2014; Brout et al., 2018; Jager et al., 2020).

For the assessment of the multidimensional experience of misophonia, Vitoratou et al. (2021b) developed a five-factor model scale known as the S-Five-E (Selective Sound Sensitivity Syndrome Scale - Experiences). The S-Five was constructed based on the responses and feedback of a large sample of English-speaking self-identified misophonic individuals, over four sampling waves. The resulting scale consists of 25-items corresponding to five dimensions: (1) *internalising appraisals* attributing blame for reactions to oneself (e.g., believing to be an unlikable or angry person), (2) *externalising appraisals* blaming other people (e.g., believing others to be rude and inconsiderate), (3) perceived emotional *threat* (e.g., feeling distress, trapped and helpless), (4) having or fearing having verbal or physical *outbursts*, and (5) the *impact* of misophonia on the ability to do things they would like to do. Along with the main scale, the S-Five has a supplementary trigger checklist (S-Five-T), which captures the emotional nature and intensity of the responses to sounds (Vitoratou et al., 2021b, 2022a). The format of the S-Five-T allows the researcher or clinician to modify the trigger sounds list and the response types, in line with changes in the growing literature on the field and individual presentations of the disorder. The five-factor model of the S-Five has been replicated in a large sample representative of the UK population (Vitoratou et al., 2022a). Excellent psychometric properties have been shown for the scale in English (Vitoratou et al., 2021b) and Mandarin (Vitoratou et al., 2022b), with cross-cultural replication of the five-factor model. A German translation, however, is still pending.

To our knowledge, the only genuine German questionnaire measuring misophonic symptoms is the Berlin Misophonia Questionnaire Revised (BMQ-R; Remmert et al., 2022). The BMQ-R reflects the proposed diagnostic criteria of misophonia by Jager et al. (2020). However, the BMQ-R is a long and comprehensive diagnostical instrument comprising 77 items. In comparison, given the S-Five’s inductive scale construction approach and resulting five core dimensions of misophonia, this scale measures typical misophonic experiences in a more efficient manner. Further, the S-Five allows to investigate the emotional nature and intensity of triggers. The strengths of the S-Five would thus certainly complement the measurement of misophonia in German samples. We therefore see merit in providing a valid German translation of the S-Five and in utilizing the strengths of both the S-Five and BMQ-R to investigate associations between misophonic symptoms.

Albeit evidence for the construct validity of the BMQ-R and the S-Five has been gathered, neither those two scales, nor any other misophonia questionnaire can be considered fully validated. Thus, construct validation plays a principal role in developing misophonia scales and in translating existing questionnaires. A widely used method for corroborating construct validity is showing evidence in line with nomological networks (Cronbach and Meehl, 1955). In nomological networks theoretical associations of constructs are to be empirically demonstrated and new constructs (e.g., misophonic symptoms) are to be placed in the proposed associational structure. To this end, hypotheses about relationships between attributes which are measured by a new instrument (e.g., the (German) S-Five) and convergent or discriminant constructs are formulated and tested. However, for relatively new constructs, such as misophonia, there is few and limited information on theoretical associations between constructs (i.e., misophonic symptoms or experiences). This does not imply the lack of a nomological network, but rather that it needs to be explored gradually. This study is a first and partially exploratory attempt to develop such a nomological network. The remainder of this introduction presents the descriptive and theoretical background for the development of the proposed nomological network of misophonia, followed by specific hypotheses and aims of the study.

A reasonable starting point for a nomological network of misophonic symptoms is the proposed diagnostic criteria put forward by Schröder et al. (2013) and revised by Jager et al. (2020). Based on a large sample of participants with misophonia, Jager et al. (2020) proposed five main symptom domains in their diagnostic criteria for clinically significant misophonia: (1) aversive emotional and physical reactions to sounds, with (2) insight into the excessive and disproportional nature of responses, (3) loss of self-control, (4) avoidance behavior, and (5) functional impairment. This description largely coincides with the recently published consensus definition of misophonia (Swedo et al., 2022). Nevertheless, several of the symptoms reported in the literature are not covered by those symptom domains, such as externalising and internalising appraisals as described by Vitoratou et al. (2021b) and Vitoratou et al. (2021a) and misophonic beliefs as described by Rosenthal et al. (2021). We therefore identified further symptom domains based on phenomenological similarities, and explored how these domains relate to different nomological aspects of misophonia. Recognition of similar psychological processes (e.g., reactions to sounds or influences on reactions; Swedo et al., 2022) and functions of symptoms (e.g., emotional regulation) is pivotal for the broadening of main symptom areas. This means grouping symptoms not necessarily by symptom type (e.g., a domain related to behavioral, cognitive, etc. symptoms), but rather by the function of the symptom (e.g., a domain for behavior used for the function of avoiding sounds or associated perceived threat, as separate from a domain for behavior used for the function of emotion regulation).

Based on the symptoms reported in contemporary misophonia literature (Jastreboff and Jastreboff, 2014; Brout et al., 2018; Potgieter et al., 2019; Jager et al., 2020; Vitoratou et al., 2021b; Swedo et al., 2022) we identified five main symptom domains: (1) misophonic appraisals, (2) misophonic emotional experiences, (3) misophonia-specific dysregulation, (4) misophonic avoidance, and (5) misophonic impairment. Critically, these symptom domains serve to give the nomological network a broader structure by clustering symptoms. In order to better understand this clustering attempt, the individual symptom domains and their associated symptoms are described in further detail below.

*Misophonic appraisals* encompass symptoms associated with the subjective meaning or evaluation placed on or knowledge about one’s own reactions to sounds and the circumstances in which they occur (i.e., attributional styles and clinical insight; Vitoratou et al., 2021b). These are meta-cognitive processes or beliefs about misophonic symptoms, rather than thoughts in response to misophonia triggers. The initial item pool for the Duke Misophonia Questionnaire (Rosenthal et al., 2021) included cognitive responses in the moment of triggers, but the items that were retained after factor analysis seemed to relate more to the state of urgency and intensity that occurs in that moment (e.g., “I would do anything to make it stop,” was retained), than to an appraisal of the situation (e.g., “They do not care how this sound affects me,” was not retained). In the symptom severity composite scale of the DMQ, these cognitive responses clustered together with other symptoms physical and emotion symptoms, not as a separate “cognitive” factor. That is, cognitions relating to the anguish of the moment were part of a dimension of physical, emotional and cognitive distress, and cognitions relating to assumptions about the moment did not seem to be part of the latent variable of misophonia symptom severity. We therefore focused this dimension on appraisals reflecting more general beliefs about the meaning of their symptoms, rather than appraisals in the moment.

The domain includes internalising and externalising appraisals (blaming for symptom experience; Rosenthal et al., 2021; Vitoratou et al., 2021b) as well as clinical insight (Jager et al., 2020; Swedo et al., 2022). Clinical insight included recognition of excess and recognition of disproportionality (e.g., see Jager et al., 2020). A broader definition of clinical insight includes the comprehension of one’s own symptoms (i.e., symptom coherence; e.g., Moss-Morris et al., 2002; Witteman et al., 2011). However, symptom coherence is a characteristic that has not been studied in the context of misophonia yet and is thus entirely exploratory in our study.

*Misophonic emotional experiences* entail all immediate emotional and physical reactions and experiences to misophonic triggers (i.e., anger, irritability, aggression, disgust, anxiety, and corresponding physical symptoms). Note that aggression entails different phenomenological aspects. We follow Buss and Perry (1992) distinguishing anger related to aggression, verbal and physical aggression as well as hostility. Physical reactions or symptoms are clustered within this domain since physical symptoms are part of the emotional misophonic response (i.e., autonomic stress response or emotional arousal; e.g., Edelstein et al., 2013). Although it has been shown that physical symptoms can be modelled as a separate misophonic factor (e.g., Dibb et al., 2021; Rinaldi et al., 2021), we do not see the benefit in separating physical reactions from the domain emotional experiences.

Misophonic emotional experiences are to be distinguished from *misophonia-specific dysregulation*, which is defined as an extension of loss of self-control (Remmert et al., 2022) as an incapability to cope with emotional experiences as well as uncontrolled behavioral manifestations for elevated levels of emotional arousal and negative affectivity. This also means disentangling various aspects of impaired self-control, including behavioral dysregulation (e.g., verbal or physical aggression), cognitive, and emotional dysregulation (i.e., loss of control over emotional experiences; e.g., Swedo et al., 2022). This domain is a category into which failed coping attempts fit (e.g., Guetta et al., 2022). It is not yet clear which domain the S-Five construct of perceived emotional *threat* fits into, as it includes items related to experiencing anxiety and distress, which may fit in the emotional experiences domain, as well as items related to feeling trapped and helpless (i.e., lack of regulative strategies to cope with misophonic experiences), which may align with the dysregulation domain.

*Misophonic avoidance* includes dysfunctional behavioral coping strategies to either prevent being exposed to misophonic sounds (anticipated avoidance) or escaping such situations (reactive avoidance; e.g., Remmert et al., 2022). Both avoidance behaviors form part of the definition of misophonia (Swedo et al., 2022). Rosenthal et al. (2021) showed that anticipated avoidance is the most prominent coping strategy before being faced with triggers, whereas reactive avoidance is the most prominent coping strategy when being triggered. Although avoidance behavior is a coping strategy, it can be distinguished from dysregulation because it serves the purpose of (re-)gaining control over the stimuli and is not the incapability to control emotional reactions. It may also include behaviors intended to prevent feared consequences of emotional dysregulation.

The fifth domain is *misophonic impairment*, which entails symptoms associated with the suffering and limitations caused by misophonic experiences (e.g., Wu et al., 2014; Jager et al., 2020; Swedo et al., 2022). Functional impact can be assigned to life domains or activities in which the impact occurs: e.g., cognitive impact, social impact, and impact on daily routine (WHO, 2001). Further, this domain entails distress as a consequence of misophonic symptoms including depressive mood and emotional burden (e.g., Jager et al., 2020; Remmert et al., 2022).

**Figure 1:**
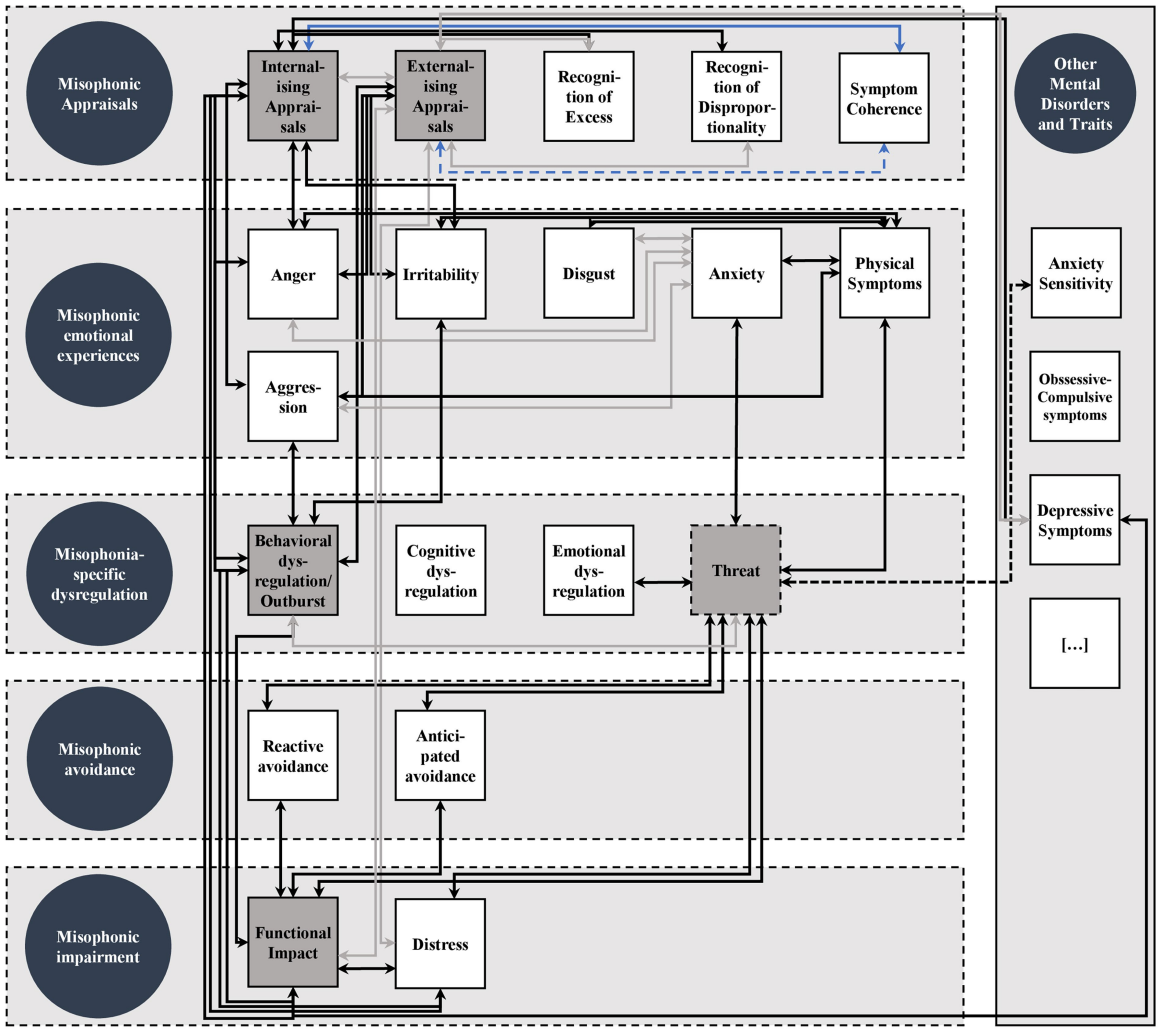
Core Nomological Network of Misophonic Symptoms. Circles represent proposed misophonic symptom domains. Squares represent specific misophonic symptoms. Grey squares represent symptoms being measured by the S-Five scales. Dashed lines around symptoms within symptoms domains shall indicate that symptom domains are not strictly separated. Strong associations are indicated by black arrows, weaker associations are grey, negative associations are blue with less negative associations in light blue, dashed arrows indicate uncertainty.

Note that this clustering of symptoms into domains is intended to facilitate the investigation of misophonic symptoms, rather than a strict classification. The domains may thus naturally overlap in some characteristics, while grouping misophonic symptoms reasonably. After having defined the broader structure of the nomological network, the following section outlines theoretical, empirical, and exploratory assumptions about how the specific symptoms are associated with each other, both within and across symptom domains (see Figure 1). Since there are 190 possible correlations between symptoms, we pragmatically concentrated on the core nomological structure, which predominantly involves symptoms being measured by the S-Five (indicated in grey boxes in the network in Figure 1). This is also due to the fact that the German S-Five is the focus of the presented studies. The proposed assumptions on associations are drawn from both misophonia research and the broader literature on mental disorders.

### Associations in the nomological network

Principally, we expect that misophonic symptoms, regardless of their proposed symptom domain, are significantly positively associated with each other. This is based on the understanding that these symptoms together constitute the higher-order construct of misophonia and are hence naturally associated with each other, which is widely empirically supported (e.g., Rinaldi et al., 2021; Rosenthal et al., 2021; Vitoratou et al., 2021a,b, 2022a,b; Remmert et al., 2022). Moreover, misophonic symptoms within the proposed symptom domains are expected to be strongly associated since they are identified on account of sharing common characteristics and functions. Any exceptions to these two principles, as well as specific hypotheses about associations between symptoms from different symptom domains, are outlined in the following sections. An additional principle of our validation approach is that explicit convergent measures (i.e., measures that exactly measure the same symptom) are assumed to correlate strongly.

#### Misophonic appraisals

Misophonic attributional styles (internalising and externalising appraisals) have been shown to correlate moderately with each other (Vitoratou et al., 2021b), which we assume to replicate in this study. Beyond this, the relationships of interest for misophonic appraisals are with clinical insight (i.e., recognition of excess and disproportionality, and symptom coherence), functional impact and distress, as well as misophonia-specific aggressive behavior (S-Five outbursts).

Individuals with misophonia often recognize that their behavior is excessive or disproportionate to the situation (Hadjipavlou et al., 2008; Jager et al., 2020; Swedo et al., 2022). Although the relationship between attributional styles and dimensions of clinical insight have not been investigated yet for misophonia, it is reasonable to assume that those recognizing their reactions as excessive or disproportionate would be more likely to attribute blame to themselves (internalising) than to other people (externalising). Critically, these relationships have been substantiated for psychiatric disorders (e.g., schizophrenia; Cotton et al., 2012) and neurodevelopmental conditions (e.g., autistic spectrum conditions; Didehbani et al., 2012). We therefore expect a higher correlation between recognition of excess or disproportionality and internalising appraisals compared with externalising appraisals. Further, it has been found that a good understanding of the nature and cause of obsessive–compulsive symptoms (i.e., symptom coherence) is associated with internal attributions, but not with external, environmental attributions (Pedley et al., 2019). Based on this, we likewise expect individuals with higher levels of symptom coherence for misophonic symptoms to be less likely to blame themselves, and instead understand that the source of the problem is not the individual, but the condition of misophonia itself. Thus, a negative correlation is expected between misophonic symptom coherence and internalising appraisals and a less negative or non-significant correlation with external appraisals.

Another characteristic of internal attribution is that it is strongly associated with depression, distress and daily impact, whereas external attribution has been shown to be less strongly associated (e.g., Peterson et al., 1981; Hu et al., 2015), which has also been shown for misophonia (Vitoratou et al., 2021b). We therefore expect strong associations to emerge between misophonic distress symptoms and functional impact with internal appraisals, but substantially less with external appraisals.

Finally, regarding the relationship with misophonia-specific aggression (outbursts), the original validation of the S-Five (Vitoratou et al., 2021b) found that the outbursts factor was moderately correlated with internalising and externalising appraisals. Surprisingly, outbursts were more strongly correlated with internalising than with externalising appraisals and both appraisal factors had low positive correlations with anger reactions to trigger sounds. While other research indicates that anger, aggression, and aggressive behavior are more frequent in those who blame others than themselves for their reactions (e.g., Averill, 1983; Quigley and Tedeschi, 1996), this appears to be have been the case with misophonia (Vitoratou et al., 2021b). We anticipate that both types of appraisals will be associated with higher levels of anger, aggression, behavioral dysregulation, and outbursts. Since irritability shares common emotional characteristics with anger and aggression (e.g., Stringaris, 2011) we assume it will also be associated with internalising and externalising appraisals.

#### Misophonic emotional experiences

Misophonia can cause a strong physical reaction (Edelstein et al., 2013; Kumar et al., 2017), which is most strongly associated with emotional reactions (Rosenthal et al., 2021). Accordingly, strong correlations between emotional misophonic responses (i.e., anger, aggression, irritability, disgust, and anxiety) and physical symptoms are assumed. Whilst anxiety may co-occur in misophonia, it is different in the psychological process compared to other emotional reactions (i.e., anger, aggression, irritability, and disgust). Anxiety is a rather anticipatory emotion caused by perceived threat whereas anger and related emotions (aggression and irritability) as well as disgust are rather reactive emotions caused by violations of personal needs, integrity or boundaries. For misophonia, anger is the most prominent reactive emotion whereas anxiety, if present, is rather anticipatory (e.g., Jager et al., 2020). Since anxiety is different from other emotional reactions in some features and does not necessarily need to co-occur, it is assumed to correlate lower (but still moderately) with other emotional reactions.

#### Misophonia-specific dysregulation

As experiencing anger, aggression and irritability when confronted with sounds might manifest in behavioral dysregulation such as aggressive outbursts (e.g., Swedo et al., 2022), these symptoms are particularly expected to correlate. Likewise, behavioral dysregulation and outbursts are likely to be related to functional impact, with this behavior naturally contributing to social conflicts and negative consequences in daily life (Wu et al., 2014). It is further hypothesized that emotional dysregulation is linked to the concept of threat as measured by the S-Five, which includes experiences of feeling trapped and helpless (i.e., expressions of dysregulated threatening emotions). Experiencing threat is conceptually and empirically related to anxiety and heightened autonomic arousal (i.e., physical symptoms; Vitoratou et al., 2021b) and therefore expected to be associated with anxiety and physical symptoms. Moreover, threat entails aspects of failed avoidance strategies in the sense that threat emerges when triggers cannot be avoided. We expect that experiencing threat motivates increased avoidance behavior in order to circumvent the feared consequences of being triggered. Thus, positive associations between threat, anxiety and avoidance strategies are expected. Threat has further been shown to be strongly correlated with functional impact (Vitoratou et al., 2021b) and is likewise expected to cause significant distress in individuals’ lives.

#### Misophonic avoidance

Experiencing threat and anxiety is generally associated with pronounced avoidance behavior causing significant distress and social isolation (Abramowitz et al., 2019). Considering the frequent reports of both anxiety and avoidance behavior in misophonia (Wu et al., 2014; Jager et al., 2020; Swedo et al., 2022), we assume that perceived threat, anxiety and avoidance behavior will be strongly correlated. Avoidance behavior can also contribute to the maintenance of symptoms (e.g., Spinhoven et al., 2017) and poor treatment outcomes, thus elevating symptom burden (e.g., Wheaton et al., 2018). Hence, we assume strong associations with functional impact.

#### Misophonic impairment

Most of the associations for symptoms from this domain have already been described in the previous sections. In summary, all misophonic symptoms being measured by the S-Five except for impact (i.e., externalising appraisals, internalising appraisal, outbursts and threat) are expected to highly correlate with symptoms from the domain misophonic impairment.

## Associations with symptoms of other mental disorders and traits

To further explore the extension of the nomological network, we also investigated associations between the S-Five and S-Five-T scores with related psychological constructs. In particular, anxiety sensitivity, which is a relatively stable trait fear of arousal-related sensations (Hovenkamp-Hermelink et al., 2019), has been shown to be related to misophonic symptoms (Cusack et al., 2018; McKay et al., 2018; Schadegg et al., 2021). Higher anxiety sensitivity was found to strengthen the relationship between misophonia and aggression (Schadegg et al., 2021). Cusack et al. (2018) found that the relationship between anxiety sensitivity and misophonia was partially mediated by obsessive–compulsive symptoms. An association between obsessive–compulsive symptoms and misophonia has also been reported elsewhere (Wu et al., 2014; Erfanian et al., 2019; Jager et al., 2020). Misophonia has been associated with symptoms of depression (Erfanian et al., 2019), particularly in relation to internalising appraisals and impact (Vitoratou et al., 2021b). This fits with the notion that internal attributional appraisals are strongly associated with depression and distress (Hu et al., 2015). Therefore, we assume high correlations between internalising appraisals and impact, and depressive symptoms. The associations between anxiety sensitivity, obsessive–compulsive symptoms and misophonic symptoms are exploratory because the associations with misophonia have only been shown for overall misophonic symptoms. However, experiencing threat when confronted with sounds entails aspects of anxiety and heightened arousal (see Misophonia-specific dysregulation), so it is likely that threat is associated with higher levels of anxiety sensitivity.

### Hypotheses

We expect to find equivalent psychometric properties for the German S-Five compared with the original version. Specifically, we hypothesize configural invariance between German-speaking and English-speaking populations, high internal reliability and high stability in time (>0.75 in agreement coefficients). We further expect to find similar intercorrelations between symptoms measured by the S-Five compared to the original validation study (Vitoratou et al., 2021b), which are outlined in Table 1. In relation to the nomological network, we outlined our hypotheses in the preceding section and summarize them in Table 2.

**Table 1:** Internal Consistencies and Intercorrelations of the S-Five from the Original Validation Study.

*Measure*	EXT	INT	IMP	OUT	THR
1. S-Five: External Appr.	(0.85)	-	-	-	-
2. S-Five: Internal Appr.	0.21	(0.88)	-	-	-
3. S-Five: *Impact*	0.29	0.50	(0.83)	-	-
4. S-Five: Outbursts	0.30	0.40	0.39	(0.84)	-
5. S-Five: Threat	0.27	0.32	0.51	0.33	(0.83)

*N* = 828. S-Five = Selective Sound Sensitivity Syndrome Scale; EXT = Externalising Appraisals, INT = Internalising Appraisals, IMP = Impact, OUT = Outbursts, THR = Threat. Cronbach’s α estimates are in parentheses on the main diagonal. The depicted correlations are rounded. All correlations are significant at *p* < 0.01.

**Table 2:** Predicted Associations between Misophonic Symptoms in the Nomological Network.

Symptom domain	Misophonic symptom (measures)	Predicted associations with other misophonic symptoms
Misophonic Appraisals	Internalising appraisals (S-Five)	Positively correlated with: externalising appraisals, recognition of excess and recognition of disproportionality (BMQ-R), functional impact, distress, irritability, anger, aggression, behavioral dysregulation/outburstsNegatively correlated with: symptom coherence (IPQ-MH)
	Externalising appraisals (S-Five)	Positively correlated with: internalising appraisals, irritability, anger, aggression, behavioral dysregulation/outburstsIn comparison with internalising appraisals less correlated with: recognition of excess and recognition of disproportionality, symptom coherence, functional impact and distress
Misophonic Emotional Experiences	Anger (BMQ-R, AQ)	Positively correlated with: physical symptoms, externalising appraisals, internalising appraisals, and behavioral dysregulation/outbursts
	Irritability (BMQ-R, BITe)	Positively correlated with: physical symptoms, externalising appraisals, internalising appraisals, and behavioral dysregulation/outbursts
	Aggression (AQ)	Positively correlated with: physical symptoms, externalising appraisals, internalising appraisals, and behavioral dysregulation/outbursts
	Anxiety (BMQ-R, STICSA)	Positively correlated with: physical symptoms and threat Less correlated with anger, irritability, aggression, and disgust than their correlations with each other
	Physical symptoms (BMQ-R, STICSA)	Positively correlated with: anger, aggression, irritability, disgust (BMQ-R, DPSS-R), anxiety and threat
Misophonia-specific Dysregulation	Behavioral dysregulation/outburst (BMQ-R, S-Five, DERS)	Positively correlated with: anger, aggression, irritability, externalising appraisals, internalising appraisals, functional impact, and distress
	Emotional dysregulation (BMQ-R, DERS)	Positively correlated with: threat
	Threat (S-Five)	Positively correlated with: emotional dysregulation, anxiety, physical symptoms, reactive avoidance, anticipated avoidance, functional impact, distress
Misophonic Avoidance	Reactive avoidance (BMQ-R, NAQ, BEAQ)	Positively correlated with: threat, functional impact
	Anticipated avoidance (BMQ-R, NAQ, BEAQ)	Positively correlated with: threat, functional impact
Misophonic Impairment	Functional impact (BMQ-R, S-Five, WHODAS 2.0)	Internalising appraisals, externalising appraisals, behavioral dysregulation/outbursts, threat, reactive avoidance, anticipated avoidance
	Distress (BMQ-R)	Internalising appraisals, externalising appraisals, behavioral dysregulation/outbursts, threat, functional impact
Other	Depressive symptoms (PHQ-9)	Positively correlated with: internalising appraisals, functional impact In comparison with internalising appraisals less correlated with: externalising appraisals.

### Aims

The study has five specific aims:

1. Provide a rigorous German translation of the S-Five and S-Five-T instruments.
2. Replicate the results from the original S-Five in German.
3. Scrutinize the psychometric properties of the scales (i.e., internal consistency, model-based reliability, and test–retest reliability as well as evidence on construct validity including the factorial structure).
4. Utilize the S-Five to investigate an associational network of misophonic symptoms to demonstrate evidence for the construct-valid measurement of misophonic symptoms using the S-Five.
5. Provide a structural and theoretical basis for further explorations of misophonic symptoms and their associations through a nomological network.

## Materials and methods

### Study overview

Two studies were conducted. The first study was part of a larger validation study that investigated a nomological network for misophonia using the responses to the 25-items of the S-Five and to the items of a new diagnostical instrument for misophonia, the Berlin Misophonia Questionnaire (BMQ-R; Remmert et al., 2022). The purpose of the second study was to provide a (partial) replication of study 1, to include the S-Five-T measure, to evaluate the stability of the German versions of the S-Five and S-Five-T, and to extend the nomological network.

### Participants

For both studies, individuals at least aged 16 or older were included in the analyses. Further eligibility criteria were not having been diagnosed with a severe learning disability or intellectual disability and having sufficient self-reported German language skills for answering the survey. Data protection guidelines were met and participants gave informed consent before completing the surveys. The studies were approved by the Ethics Committee at the Department of Education and Psychology of the Freie Universität Berlin, Germany (document number: 029/2020) and by the PNM Research Ethics Panel, King’s College London (RESCM-19/20-11,826).

In study 1, we further administered items assessing participants’ attention in line with DeSimone et al. (2015) and chose 80% correct answers as an inclusion cut-off. To check for aberrant response behavior we calculated a response pattern index as proposed by Meade and Craig (2012) excluding participants with more than 30% consecutive equal answers. The first study aimed at the evaluation of the dimensionality of the S-Five. Forero et al. (2009) suggested a sample size of *N* > 200–500 for using latent trait models for ordinal data using the WLSMV estimator. We collected data from 952 individuals, of which *N* = 639 (67.12%) completed the S-Five and met the inclusion criteria. For study 2, we recruited 322 participants, of which *N* = 235 (73.0%) met the inclusion criteria and completed at least the S-Five. The second study focused on the translation of the S-Five-T complementary trigger checklist along with providing a confirmation dataset for the factor structure of the S-Five scale and evaluating stability.

Both studies were conducted using social media platforms in Germany (e.g., Facebook and Instagram) as well as university mailing lists. Groups with individuals identifying as having misophonia as well as unspecific recruitment groups and groups with individuals suffering from any form of impaired hearing or disorders related to hearing (e.g., tinnitus, hyperacusis, etc.) were included in the sampling frame. The recruitment language was German. As an incentive participants could participate in a lucky draw for 10 × 5 Euro Amazon voucher and psychology students received course credit. In the second study motivation was provided in terms of a lucky draw for 25 × 20 Euro amazon vouchers. A test-retest study was conducted two to 4 weeks later.

### Translation procedure

The translations of the scales from their respective language (i.e., Polish or English) to German was conducted by applying the TRAPD procedure (Harkness, 2003). TRAPD is an acronym for the following steps ensuring the quality of questionnaire translation: translation, review, adjudication, pretesting, and documentation. Two translators, who are fluent or native speakers of the respective languages, independently translated the items of each scale. The translated items were then reviewed with the translators and authors of the study (three of whom are German native speaker and fluent in English). Objects of the review were content, wording, and authenticity (i.e., evaluation of how natural or native the translation is) of the items. In this part, alterations of items were implemented if indicated.

### Measures

All measures are described in detail below. For both studies, three scales measuring aspects of misophonia were administered: S-Five, BMQ-R, and MisoQuest.

For study 1, non-misophonia specific scales were administered, each with its instructions contextualized for the respondent to answer in relation to misophonia. At the beginning of the study, participants were asked to think about the sounds that bothered them most or, if not applicable, about typical misophonic sounds (i.e., eating, swallowing, and sniffing) and were instructed to consider either the presence or impact of those sounds in relation to each scale. For instance, we added the accessory sentence: “[…] when you are confronted with bothersome sounds.” This procedure aimed at minimizing between-person variability and within-person inconsistency due to thinking about different contexts when giving a response and thus aimed at increasing validity (cf. Lievens et al., 2008). The scales contextualized for misophonic sounds in study 1 were the Aggression Questionnaire (AQ), Brief Irritability Test (BITe), State–Trait Inventory for Cognitive and Somatic Anxiety (STICSA), Difficulties in Emotion Regulation Scale (DERS), Noise Avoidance Questionnaire (NAQ), Brief Experiential Avoidance Questionnaire (BEAQ), World Health Organization Disability Assessment Schedule 2.0 (WHODAS 2.0), Illness Perception Questionnaire Mental Health (IPQ-MH). Note that items from each instrument were administered randomly in blocks. For the BMQ-R, items from each symptom area were presented in randomized blocks (see Remmert et al., 2022).

For study 2, non-misophonia-specific scales were not contextualized for misophonia, because we aimed at investigating associations with adjacent clinical constructs not only limited to misophonic contexts. Further, the three constructs are not described as misophonic symptoms, so it is not reasonable to contextualize them accordingly. These were the Anxiety Sensitivity Index 3 (ASI-3), Patient Health Questionnaire 9 (PHQ-9), and the Dimensional Obsessive–Compulsive Scale (DOCS). Additionally, the S-Five-T was included in study 2, but not in study 1. For the S-Five, the order of questions (five S-Five items per question), and the order of item presentation in each question were randomized. Answering the S-Five-T trigger checklist was optional and participants were given the opportunity to skip to the following section after each trigger sound presented. This was done to minimize the potential distress and discomfort experienced when reading about misophonic triggers. Moreover, participants were randomly evenly allocated to either the BMQ-R or MisoQuest. The order of presentation for the BMQ-R items was randomized, as well as the order of the PHQ-9, DOCS, and ASI-3 thereafter. The three non-misophonia scales were optional. The links for the test-retest were sent out two to 4 weeks after initial participation in the survey. The follow-up survey contained the S-Five, S-Five-T, and basic demographic data such as a unique participant identification number and age.

#### Measures of misophonia

The Selective Sound Sensitivity Syndrome Scale (S-Five; Vitoratou et al., 2021b) is a self-report instrument measuring misophonic symptoms which consists of 25 items corresponding to five subscales: internalising appraisals, externalising appraisals, perceived threat, outbursts, and impact. Items are rated on an 11-point rating scale (0 = not at all true to 10 = completely true). A supplementary trigger checklist, the S-Five-T, consists of 37 misophonic triggers and both the emotional response (e.g., anger and disgust) and the intensity (from 0 to 10) of the response to triggers. Three indices can be derived: Trigger Count (i.e., number of triggers; TC), Frequency/Intensity of Reactions Score (i.e., total value of the intensity of triggers; FIRS), Relative Intensity of Reactions Score (i.e., intensity of reactions relative to the number of triggers; RIRS). The German and English S-Five can be found in the Supplementary material.

The Berlin Misophonia Questionnaire Revised (BMQ-R; Remmert et al., 2022) is a multidimensional diagnostical instrument for measuring misophonic symptoms. It consists of 15 symptom-oriented scales (excluding scales on anticipated reactions to sounds) which can be assigned to their corresponding diagnostic criteria by Jager et al. (2020). The scales have been shown to be reliable with McDonald’s ω ranging from.72 to.94. Results from latent variable models as well as correlations with convergent and discriminant measures give substantive evidence regarding construct validity. In total, 67 items were used, which are rated on a 6-point rating scale (0 = does not apply at all to 5 = completely applies).

MisoQuest^1^ (Siepsiak et al., 2020) is a unidimensional self-report instrument of misophonia with 14 items, rated on a 5-point Likert scale (1 = strongly disagree to 5 = strongly agree). The instrument was translated from Polish into German.

#### Emotion states and dispositions

The Aggression Questionnaire (AQ; Buss and Perry, 1992) comprises four dimensions: (1) physical aggression, (2) verbal aggression, (3) anger and (4) hostility with 29 items being rated on a 4-point rating scale (1 = does not apply to 4 = fully applies). We used the German version of the Aggression Questionnaire (Werner and von Collani, 2004)^2^ in an optimized version for the measurement of misophonia (see Remmert et al., 2022).

The Disgust Propensity and Sensitivity Scale Revised^3^ (DPSS-R; Cavanagh and Davey, 2000) reduced-item version (Fergus and Valentiner, 2009), is a measure of disgust encompassing the dimensions disgust propensity and disgust sensitivity. The items measure the frequency of physical and emotional symptoms of disgust which are rated on a 5-point rating scale (1 = never to 5 = always). For this study, only the disgust propensity (DP) items were used (six items), which measure how easily an individual is repulsed. The instrument was translated from English into German.

The State–Trait Inventory for Cognitive and Somatic Anxiety^3^ (STICSA; Ree et al., 2008) measures dimensions of state and trait anxiety. Only the two trait scales were used for the present study, which capture a predisposition to experience anxiety in response to certain types of stressors, namely cognitive (10 items) and somatic (11 items) stressors. The items are rated on a 4-point rating scale (1 = not at all to 4 = very much so). The instrument was translated from English into German and optimized for the measurement of misophonia (see Remmert et al., 2022).

The Anxiety Sensitivity Index (ASI-3; Taylor et al., 2007) consists of 18 items that assess anxiety sensitivity, that is fear of anxiety-related sensations. It consists of three subscales: physical, cognitive, and social concerns. Responses are given on a 5-point rating scale from 0 = do not agree at all to 4 = fully agree. The German version was developed by Kemper et al. (2011).

The Brief Irritability Test^4^ (BITe; Holtzman et al., 2015) is a 5-item measure of irritability in the last 2 weeks. Items are rated on a 6-point rating scale (1 = never to 6 = always). We used the German version by Krey (2017).

The Dimensional Obsessive–Compulsive Scale (DOCS; Abramowitz et al., 2010) is a 20-item measure of obsessive–compulsive disorder. There are four categories of concerns: contamination, responsibility for harm, unacceptable thoughts, and “just right” concerns (denoted as symmetry). For each category there are five questions (rated from 0 to 4), asking about time occupied, avoidance behaviors, associated distress, functional impairment, and resistance to obsessions and compulsions. The German version by Fink-Lamotte et al. (2020) ^5^ was used.

The Patient Health Questionnaire (PHQ-9; Kroenke et al., 2001) is a 9-item measure of symptoms of depression. Respondents answer how often they were bothered by each symptom in the past 2 weeks, on a 4-point rating scale from 0 = not at all to 3 = almost every day. We used the German version by Gräfe et al. (2004).^6^

#### Emotion regulation

The Difficulties in Emotion Regulation Scale (DERS; Gratz and Roemer, 2004) is a measure of emotion regulation which consists of six subscales. For this study, we chose the following three subscales: (1) impulse control difficulties, (2) difficulties engaging in goal-oriented behavior, and (3) limited access to emotion regulation. These subscales consist of 19 items in total, of which 15 were chosen regarding their content validity to match the intended validation purpose. The items are rated on a 5-point rating scale regarding the experienced frequency (1 = almost never (0–10%) to 5 = almost always (91–100%)). The German version by Gutzweiler and In-Albon (2018)^4^ was used.

#### Avoidance behavior

The Noise Avoidance Questionnaire^5^ (NAQ; Bläsing and Kröner-Herwig, 2012) is a German self-report instrument measuring sound avoidance in daily life. It comprises 25 items of which 10 items describe specific situations that might be avoided. The remaining 15 items refer to specific behaviors related to sound avoidance. The more behavior-oriented items were chosen which are rated on a 5-point rating scale (1 = never to 5 = very often/always). We could not obtain the German items, so that the English items were translated. Since the statements are short and concise, we do not expect compromising effects due to translation, however, we optimized the item selection for the measurement of misophonia (see Remmert et al., 2022).

The Brief Experiential Avoidance Questionnaire (BEAQ; Gámez et al., 2014) is a 15-item measure of avoidance behavior. For this study, items from the original Behavioral Avoidance subscale of the German version (Böge et al., 2020)^7^ were relevant as they reflect situational avoidance of physical distress. Items are rated on a 6-point rating scale (1 = strongly disagree to 6 = strongly agree).

#### Impairment

The World Health Organization Disability Assessment Schedule 2.0^8^ (WHODAS 2.0; Üstün et al., 2010) is a clinical instrument based on the International Classification of Functioning, Disability and Health (ICF; WHO, 2001) which measures the impact of a given health condition in six domains of life: Cognition, mobility, self-care, getting along, life activities, and participation. Since mobility and self-care appear to be irrelevant for misophonia, these domains were not administered. The German self-report 36-item version (27 items after discarding the two domains) was optimized for the measurement of misophonia (see Remmert et al., 2022). Items are rated regarding the extent of difficulty individuals have doing the presented activities using a 5-point rating scale (1 = none to 5 = extreme or cannot do).

#### Clinical insight

Illness Perception Questionnaire Mental Health (IPQ-MH; Witteman et al., 2011) is an adapted version of the Illness Perception Questionnaire Revised (IPQ-R; Moss-Morris et al., 2002) measuring an individual’s perception of their mental health problem. Only the coherence subscale (five items), measuring the extent of an individual’s understanding of their mental health problem, was used for this study. The items are rated on a 5-point rating scale (1 = strongly disagree to 5 = strongly agree), with a higher score indicating more symptom coherence. The German version of the IPQ-R^9^ (Gaab et al., 2008) was used and adapted in line with Witteman et al. (2011) by replacing the term ‘illness’ with ‘problem’ in each item. There was one item from the coherence subscale of the IPQ-R that was removed from the scale for the final version of the IPQ-MH (“the symptoms of my condition are puzzling to me”). We included it in our survey as it had appeared in the German version of the IPQ-R and after initial psychometric examination showed a good fit, we retained the item in the measure.

### Statistical analyses

The subscales of the S-Five were jointly modelled in a confirmatory factor analysis (CFA) according to the specified measurement model by Vitoratou et al. (2021b). Therefore, we specified a correlated first-order factor model. Measurement models of the validation instruments were specified according to the original factor structure, but sometimes with an optimized set of items which adequately fit the measurement of misophonia (see Remmert et al., 2022). For the DERS scales we specified an S•I-1 model (Eid et al., 2017) with one item as the reference item (general dysregulation) and the other items as specific factors (dysregulation facets), which is different from the original. This procedure allows us to investigate associations of misophonic symptoms with general dysregulation and its facets rather than with the facets alone.

Non-normality and categorical indicators were taken into account using the weighted least square mean and variance adjusted (WLSMV; Muthén et al., 1997) estimator with ordered categories. For the S-Five (continuous indicators) we used maximum likelihood estimation with robust (Huber-White) standard errors (Huber, 1967; White, 1980). Item omissions were addressed using full information maximum likelihood estimates.

Model fit was evaluated by using absolute and relative fit indices. Namely, the exact relative χ^2^ (that is the ratio of the χ^2^ over the degrees of freedom) with values ranging from 2 (Hoelter, 1983; Ullman, 2001) to < 5 (Wheaton et al., 1977; West et al., 2012) indicating adequate fit (Schermelleh-Engel et al., 2003), the Root Mean Squared Error of Approximation (RMSEA) with values close to 0.06 indicating adequate fit (Hu and Bentler, 1999), the Standardized Root Mean Square Residual (SRMR) with values close to 0.08 indicating good fit (Hooper et al., 2008), McDonald’s Centrality Index (Mc) close to 0.90 (Hu and Bentler, 1999); as well as Comparative Fit Index (CFI) and Tucker Lewis Index (TLI) close to 0.97 (Schermelleh-Engel et al., 2003). The Expected Cross Validation Index (ECVI) was used to compare non-nested models (Browne and Cudeck, 1989).

The multiple indicator multiple causes model (MIMIC; Joreskog and Goldberger, 1975; Muthén, 1979) was used to assess measurement invariance in relation to gender and age. An item was regarded as non-invariant when the effect of the exogenous variable (age or gender) on the item directly (hereafter direct effect or de) was statistically significant.

Internal consistency was estimated with model-based McDonald’s ω (McDonald, 1999). The test-retest reliability was evaluated using the intraclass correlations coefficient (ICC; Shrout and Fleiss, 1979). ICC values > 0.75 are interpreted as good reliability, according to Koo and Li (2016).

Correlations were interpreted in line with Cohen (1988); i.e., *r* = |0.10|, *r* = |0.30|, *r* = |0.50| are considered weak, moderate and strong, respectively. Differences between correlations were statistically compared using Fisher’s z-test of dependent correlation or between an empirical and a hypothesized correlation (Fisher, 1956). We applied Bonferroni correction to significance-level α in order to address α-error inflation due to multiple testing (Bonferroni, 1936) and further decided to be as conservative as possible, thus correcting for all calculated correlations per study. In study 1 we calculated 820 correlations and therefore α = 0.00006 and for study 2 we calculated 465 correlations and therefore α = 0.0001. Additionally, we corrected for hypothesized correlation comparisons. For the 17 comparisons in study 1 α = 0.0029 and for the 17 comparisons in study 2 α = 0.0029. Ten comparisons between independent samples were tested using Fisher’s z-test of correlations in two independent samples (Fisher, 1956) with a corrected α = 0.005. Note that due to dropouts (respectively pairwise complete analyses) the sample sizes of dependent comparisons between correlation may vary within both studies, so we always selected the smallest overlapping sample size and still counted all comparisons within each study to adjust alpha-inflation, which is the most conservative method. The statistical software of Stata 16 (StataCorp, 2019), Mplus 8 (Muthen and Muthén, 1998-2017), and the “lavaan” package (version 0.6-9; Rosseel, 2012) in R (R Core Team, 2017) were used to carry out the analysis.

## Results

### Descriptive indices

In sample 1 with *N* = 639, most participants (86.2%) were female, and two individuals indicated non-binary gender. The mean age was M = 34.28 years (SD = 11.52, range 16 to 69). Approximately one-third (32.7%) were students. Further, one-third of the sample had a university degree and 46.7% had at least a college entrance qualification. A majority had a partner or was married (65.1%), whereas 33.9% did not have a partner or was living separated. Almost a third of the sample (30.3%) was either part-time or marginally employed, 37.3% was full-time employed, and 13.3% unemployed. About half of the sample (47.5%) fulfilled the diagnostic criteria by Jager et al. (2020) (with 24.6% having severe symptoms) as classified by the BMQ-R.^10^ According to the S-Five total score cut-off (i.e., total score of 87 or higher; *cf.* Vitoratou et al., 2022a), more than half of the sample (57.4%) had significant misophonia.

In sample 2, with *N* = 235, the majority of participants was female (85.1%), with two participants identifying as non-binary. The mean age was 35.8 years (SD = 11.8, range 19 to 80). The majority of the sample (95.3%) reported living in Germany or another German-speaking country (Germany 86.0%, Austria 5.5%, Switzerland 3.0, 5.9% rest of world). In terms of educational attainments, 6.4% had up to high school, 47.7% reported having done apprenticeships, 26.8% undergraduate degree, 14.5% postgraduate degree, and 4.7% doctoral or similar. Significant misophonia as indicated by the S-Five total cut-off was observed for 58.3% of the sample.

### Structural validity and measurement invariance

**Figure 2:**
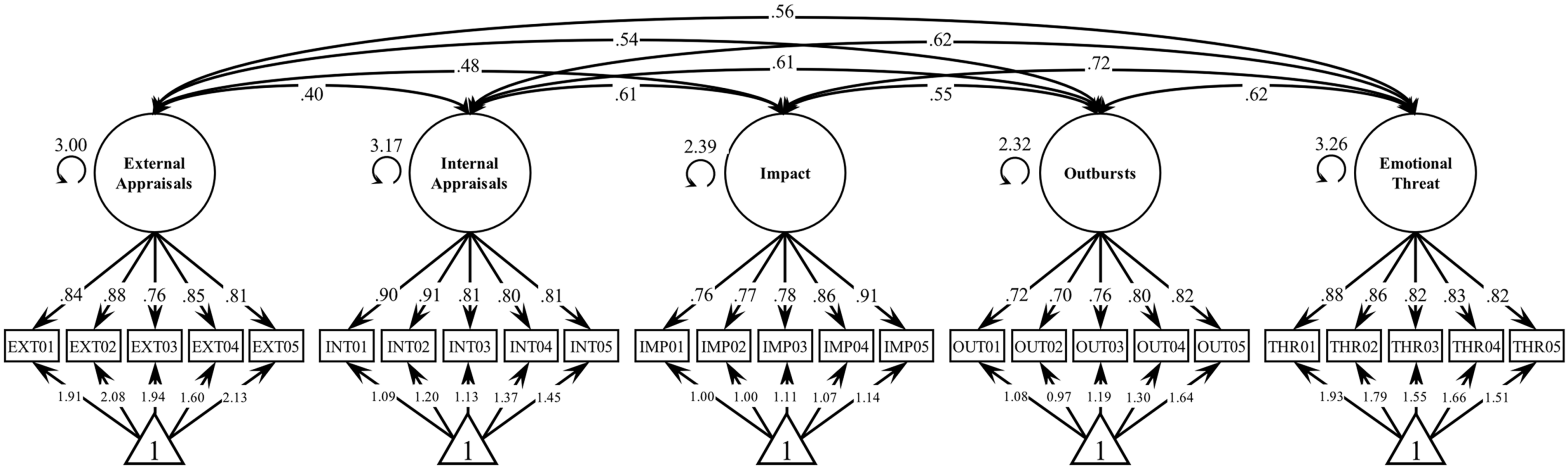
First-Order Factor Model of S-Five Scales. *N* = 639. All factor loadings, intercepts, and correlations are significant at *p* < 0.00006. Factor loadings and intercepts are completely standardized (both latent and observed variables). Unstandardized factor standard deviations are shown next to the latent variables.

The five-factor correlated model showed adequate fit to the data in both the first [χ^2^(265) = 850.93, *p* < 0.001, rel. χ^2^ = 3.21, CFI = 0.94, TLI = 0.93, RMSEA = 0.07 [0.06–0.07], SRMR = 0.05, Mc = 0.55, ECVI = 1.87] and the second sample [χ^2^(265) = 452.15, *p* < 0.001, rel. χ^2^ = 1.71, CFI = 0.94, TLI = 0.94, RMSEA = 0.06 [0.05–0.06], SRMR = 0.05]. An outline of the estimated model in sample 1 is shown Figure 2.

Measurement invariance was explored with respect to gender and age, each adjusted for the other and the levels of the five factors. In sample 1, five items were directly affected from age but with negligible effect sizes (INT01: de = −0.018, *p* = 0.017; INT02: de = −0.020, *p* = 0.005, INT04: de = 0.020, *p* = 0.042, IMP03: de = 0.032, *p* = 0.001, IMP02: de = 0.670, *p* = 0.017). Two items were also affected from gender adjusted for age and the five misophonic dimensions of the misophonic experience (INT05: de = 0.698, *p* = 0.001; OUT03 de = 0.670, *p* = 0.017), with however less than one unit of effect on a 0 to 10 scale. Similar results emerged in the second sample, with three significant effects emerging for either age (INT02: de = −0.037, *p* = 0.002, INT04: de = −0.034, *p* = 0.007, OUT04: de = −0.032, *p* = 0.017) and gender (EXT01: de = 1.001, *p* = 0.013; IMP01: de = 1.068, *p* = 0.002; IMP03: de = 1.588, *p* < 0.001), with low magnitudes in either case.

**Figure 3:**
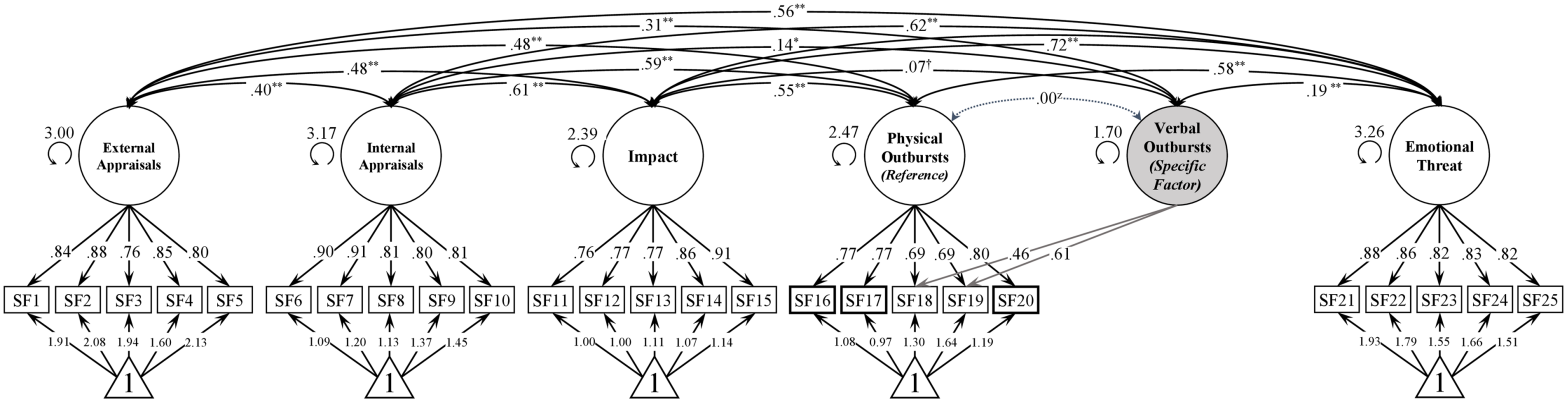
Bifactor S-1 Model of S-Five Scales. *N* = 639. Factor loadings and intercepts are completely standardized (both latent and observed variables). Unstandardized factor standard deviations are shown next to the latent variables. ^z^The correlation is per definition set to zero. ^†^n.s., ^*^p < 0.05, ^**^p < 0.01.

An alternative bifactor S-1 model (Eid et al., 2017) was also fitted as from a theoretical perspective, the outburst factor comprises both verbally as well as physically aggressive behaviors. The bifactor S-1 model maintains a general outburst factor but takes the implied two-dimensionality of outbursts into account. The model was specified with physically aggressive behavior as the reference facet (G-factor) and verbally aggressive behavior as the specific factor yielding a model with good fit [χ^2^(4) = 11.84, *p* < 0.05, rel. χ^2^ = 2.96, CFI = 0.99, TLI = 0.98, RMSEA = 0.07 [0.03–0.12], SRMR = 0.02, Mc = 0.99, ECVI = 0.08]. Likewise, an adapted five-factor correlated model integrating the presented bifactor S-1 approach for the factor outbursts demonstrated good fit [χ^2^(259) = 714.73, *p* < 0.001, rel. χ^2^ = 2.76, CFI = 0.96, TLI = 0.95, RMSEA = 0.06 [0.05–0.06], SRMR = 0.05, Mc = 0.63, ECVI = 1.63]. Model comparison using a likelihood ratio test yielded a significantly better model fit of the bifactor S-1 model [Δχ^2^(6) = 115.29, *p* < 0.001, ΔCFI = 0.01, ΔRMSEA = 0.01]. For an outline of the alternative bifactor S-1 model see Figure 3.

### Internal consistency and intercorrelations of the S-Five

In both samples, the estimated internal consistencies of the factors were good to excellent, according to McDonald’s ω (ω ranged from 0.86 to 0.93; see Table 3). Descriptively, we found similar internal consistencies compared with the original validation study, except for impact and perceived threat, which were found to be slightly higher in our studies. In Study 1, the factor intercorrelations ranged from *r* = 0.40 to *r* = 0.72 with threat and impact being highest correlated (Table 1). Similarly, in study 2 the factor intercorrelations ranged from *r* = 0.51 to *r* = 0.79, with threat and impact again being most strongly correlated. All intercorrelations are significantly higher than in the original validation study (*p* < 0.005 for all comparisons), but we found almost the same correlational pattern. An exception was perceived threat which was comparably higher correlated with internalising appraisals and outbursts than other factors were correlated with internalising appraisals and outbursts. This aligns with the fact that perceived threat and outbursts were in general unproportionally highly correlated with other factors when compared to the original validation study (differences between 0.16 and 0.36).

**Table 3:** Means and standard deviations, latent (Study 1) and Spearman’s (Study 2) intercorrelations, and reliability estimates for the S-Five.

*Measure*	Min–Max	Study 1		Study 2		EXT	INT	IMP	OUT	THR
		M	SD	M	SD					
1. S-Five: External Appr.	0–50	28.97	15.26	27.31	15.70	(0.92/0.92)	0.51	0.59	0.57	0.58
2. S-Five: Internal Appr.	0–50	17.78	15.96	16.85	14.74	0.40	(0.93/0.88)	0.64	0.72	0.73
3. S-Five: *Impact*	0–50	12.19	13.83	14.76	14.08	0.48	0.61	(0.90/0.91)	0.65	0.79
4. S-Five: Outbursts	0–50	16.20	13.91	16.50	13.97	0.54	0.61	0.55	(0.87/0.86)	0.69
5. S-Five: Threat	0–50	26.85	16.55	28.98	17.28	0.56	0.62	0.72	0.63	(0.92/0.89)

*N* = 639 (Study 1); *N* = 235 (Study 2). Cells below the diagonal represent latent intercorrelations for Study 1; Cells above the diagonal represent Spearman’s correlation coefficients (ρ) for Study 2. S-Five = Selective Sound Sensitivity Syndrome Scale; EXT = *Externalising* Appraisals, INT = *Internalising* Appraisals, IMP = *Impact*, OUT = Outbursts, THR = Threat. McDonald’s ω (McDonald, 1999) based on the respective confirmatory factor analyses are in parentheses on the main diagonal (on left = Study 1, right = Study 2). All correlations for study 1 were significant at *p* < 0.00006 and for study 2 at *p* < 0.0001 (Bonferroni-corrected significance level, respectively). M = mean, SD = standard deviation; Min = scale minimum, Max = scale maximum; *N* = 633-636 (Study 1); *N* = 235 (Study 2). Means were calculated for manifest sum scores of the respective scale.

### Test–retest reliability (study 2)

The S-Five items and scores all showed excellent agreement across the test and retest (*N* = 52), with ICC ≥ 0.86 in all cases and ICC = 0.90 for the total S-Five score. Similarly for the S-Five-T trigger scores, agreement was excellent with ICC ≥ 0.84 in all cases and ICC = 0.90 for the TC, FIRS, and RIRS.

### Nomological network of misophonic symptoms: Construct validity

**Figure 4:**
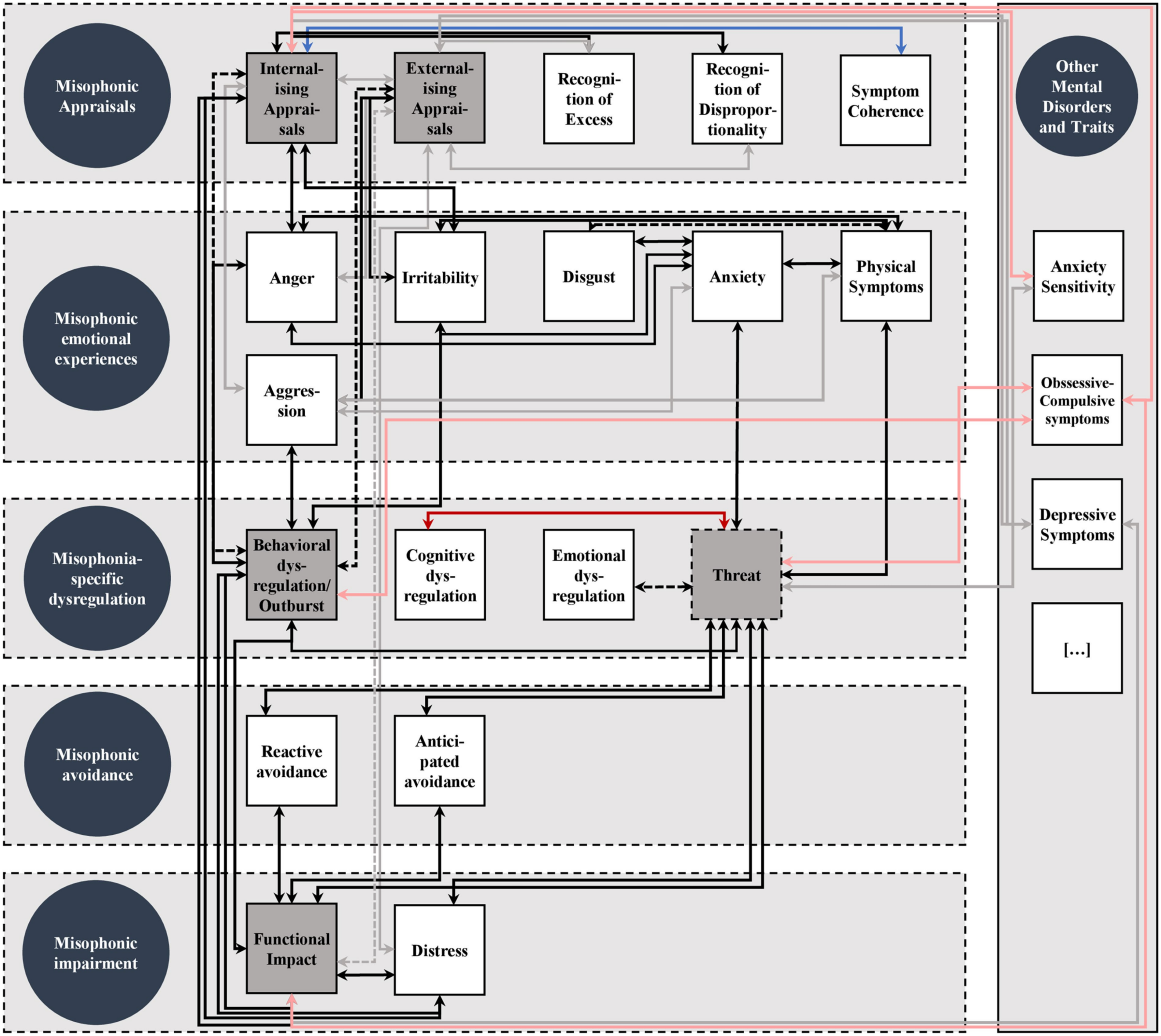
Empirical Nomological Network of Misophonic Symptoms. Circles represent proposed misophonic symptom domains. Squares represent specific misophonic symptoms. Grey squares represent symptoms being measured by the S-Five scales. Dashed lines around symptoms within symptoms domains shall indicate that symptom domains are not strictly separated. Strong associations are indicated by black arrows, weaker associations are grey, negative associations are blue with less negative associations in light blue, exploratory associations are red with weaker associations in light red, dashed arrows indicate uncertainty due to mixed results.

In this section, we report results regarding the proposed nomological network of misophonic symptoms for each symptom domain. In each section, we first describe associations within the respective symptom domain followed by associations between different domains and associations with symptoms of other mental disorders and traits. We additionally report associations between misophonic trigger scores and between S-Five scales and overall misophonic symptoms at the end of this section. An updated version of Figure 1 depicting the empirical nomological network can be found in Figure 4.

#### Misophonic appraisals

Internalising and externalising appraisals were moderately to highly positively correlated (Table 3). For internalising appraisals, the correlations with recognition of disproportionality and excess were higher than the moderate correlations emerging with externalising appraisals (*p* < 0.0029 for all four comparisons; Table 4). Coherence of misophonic symptoms (IPQ-MH), i.e., the level of comprehension regarding misophonic symptoms, moderately negatively correlated with internalising appraisals, whereas externalising appraisals were not significantly associated with symptom coherence and were further less negatively correlated compared to internalising appraisals (*p* < 0.0029; Table 5).

**Table 4:** Means and standard deviations, latent (Study 1) and Spearman’s (Study 2) intercorrelations, and reliability estimates of the BMQ-R symptom part, and MisoQuest.

*Measure*	Study 1									Study 2								
	M	SD	Min–Max	EXT	INT	IMP	OUT	THR	ω	M	SD	Min–Max	EXT	INT	IMP	OUT	THR	ω
BMQ: Anger	13.42	5.74	0–20	0.59	0.60	0.58	0.74	0.72	0.90	12.22	6.95	0–20	0.62	0.63	0.65	0.70	0.75	0.92
BMQ: Irritation	15.39	4.18	0–20	0.59	0.59	0.67	0.58	0.81	0.77	14.08	6.15	0–20	0.60	0.54	0.60	0.56	0.75	0.90
BMQ: Disgust	10.27	6.50	0–20	0.46	0.38	0.38	0.40	0.52	0.92	10.56	6.79	0–20	0.63	0.69	0.50	0.63	0.59	0.91
BMQ: Anxiety	6.19	6.01	0–20	0.41	0.49	0.63	0.48	0.77	0.88	7.17	6.19	0–20	0.54	0.52	0.73	0.63	0.75	0.87
BMQ: Physical	7.26	4.87	0–15	0.50	0.62	0.64	0.64	0.81	0.85	8.47	4.81	0–15	0.53	0.54	0.69	0.67	0.76	0.83
BMQ: R. Disp.	12.66	6.14	0–20	0.33	0.65	0.47	0.53	0.61	0.90	9.75	6.92	0–20	0.34	0.59	0.34	0.49	0.39	0.93
BMQ: R. Exc.	11.06	6.45	0–20	0.47	0.70	0.62	0.67	0.78	0.91	10.07	6.66	0–20	0.52	0.69	0.63	0.72	0.72	0.92
BMQ: G. Dys.	10.96	5.27	0–20	0.38	0.49	0.51	0.63	0.64	0.90	-	-	-	-	-	-	-	-	-
BMQ: B. Dys.	8.81	5.54	0–20	0.47	0.65	0.52	0.84	0.62	0.87	8.48	6.54	0–20	0.50	0.69	0.56	0.75	0.63	0.89
BMQ: C. Dys.	14.95	4.77	0–20	0.49	0.54	0.57	0.56	0.72	0.90	-	-	-	-	-	-	-	-	-
BMQ: E. Dys	10.95	5.62	0–20	0.51	0.64	0.67	0.65	0.86	0.87	-	-	-	-	-	-	-	-	-
BMQ: Re. Av.	13.71	4.82	0–20	0.50	0.52	0.67	0.50	0.76	0.71	12.40	5.80	0–20	0.57	0.55	0.54	0.63	0.71	0.80
BMQ: Ant. Av.	10.77	6.79	0–20	0.48	0.46	0.70	0.43	0.69	0.93	9.43	6.92	0–20	0.58	0.47	0.82	0.62	0.71	0.94
BMQ: Distress	15.52	7.95	0–25	0.55	0.72	0.76	0.66	0.85	0.94	15.36	8.22	0–25	0.43	0.58	0.64	0.59	0.74	0.94
BMQ: Fun. Imp.	11.67	9.99	0–35	0.51	0.63	0.85	0.61	0.83	0.91	14.28	10.50	0–35	0.57	0.58	0.83	0.72	0.75	0.93
MisoQuest	33.78	14.65	0–56	0.62	0.71	0.73	0.70	0.88	0.93	32.09	16.18	0–56	0.53	0.71	0.78	0.71	0.89	0.96

*N* = 609-616 (Study 1); *N* = 102-108 (Study 2). S-Five = Selective Sound Sensitivity Syndrome Scale; EXT = *Externalising* Appraisals, INT = *Internalising* Appraisals, IMP = *Impact*, OUT = Outbursts, THR = Threat; BMQ: Berlin Misophonia Questionnaire Revised; R. Disp. = Recognition of Disproportionality; R. Exc. = Recognition of Excess; G. Dys. = General Dysregulation; B. Dys. = Behavioral Dysregulation; C. Dys. = Cognitive Dysregulation; E. Dys. = Emotional Dysregulation; Re. Av. = Reactive Avoidance; Ant. Av. = Anticipatory Avoidance; Fun. Imp. = Functional Impact. McDonald’s ω based on the respective confirmatory factor analyses are in parentheses on the diagonal. All correlations in study 1 were statistically significant at *p* < 0.00006 (Bonferroni-corrected significance level in study 1). All correlations in study 2 were statistically significant at *p* < 0.0001 (Bonferroni-corrected significance level in study 2). M = mean, SD = standard deviation, Min = scale minimum, Max = scale maximum; *N* = 633-636 (Study 1); *N* = 102-108 (Study 2). Means were calculated for manifest sum scores of the respective scale.

**Table 5:** Latent (Study 1) and Spearman’s (Study 2) intercorrelations of the S-Five with AQ, BITe, STICSA, DERS, NAQ, BEAQ, IPQ-MH, WHODAS 2.0, PHQ-9, ASI-3, and DOCS.

*Measure*	Study 1									Study 2								
	M	SD	Min–Max	EXT	INT	IMP	OUT	THR	ω	M	SD	Min–Max	EXT	INT	IMP	OUT	THR	ω
AQ: Anger	8.64	4.34	0–18	0.46^I^	0.56^I^	0.46^I^	0.67^I^	0.49^I^	0.82	-	-	-	-	-	-	-	-	-
AQ: Verbal Aggression	2.77	1.96	0–9	0.39^I^	0.33^I^	0.36^I^	0.46^I^	0.32^I^	0.63	-	-	-	-	-	-	-	-	-
AQ: Physical Aggression	2.84	2.87	0–18	0.30^I^	0.27^I^	0.26^I^	0.60^I^	0.24^I^	0.71	-	-	-	-	-	-	-	-	-
AQ: Hostility	6.59	4.32	0–18	0.40^I^	0.52^I^	0.47^I^	0.43^I^	0.43^I^	0.79	-	-	-	-	-	-	-	-	-
BITe: Irritability	12.09	6.11	0–25	0.48^I^	0.58^I^	0.53^I^	0.57^I^	0.66^I^	0.91	-	-	-	-	-	-	-	-	-
STICSA: Cognitive	11.30	7.09	0–27	0.45^I^	0.64^I^	0.61^I^	0.47^I^	0.67^I^	0.91	-	-	-	-	-	-	-	-	-
STICSA: Somatic	8.12	6.37	0–27	0.44^I^	0.52^I^	0.58^I^	0.53^I^	0.70^I^	0.90	-	-	-	-	-	-	-	-	-
DERS: Impulse Control	4.42	2.98	0–12	−0.01^†^	0.01^†^	0.08^†^	0.23^**^	−0.07^†^	0.70	-	-	-	-	-	-	-	-	-
DERS: G-O Behavior	7.32	3.51	0–12	0.08^†^	−0.04^†^	0.20^**^	−0.11^†^	0.14^**^	0.90	-	-	-	-	-	-	-	-	-
DERS: Emot. Dys.	13.13	8.14	0–32	0.11^*^	0.18^**^	0.25^I^	0.00^†^	0.15^**^	0.91	-	-	-	-	-	-	-	-	-
DERS: Gen. Dys.	26.39^a^	14.04^a^	0–60	0.40^I^	0.60^I^	0.49^I^	0.59^I^	0.69^I^	0.83^b^	-	-	-	-	-	-	-	-	-
NAQ: Noise Avoidance	10.78	9.10	0–44	0.50^I^	0.51^I^	0.77^I^	0.49^I^	0.66^I^	0.91	-	-	-	-	-	-	-	-	-
BEAQ: Behav. Avoidance	8.71	4.84	0–22	0.44^I^	0.40^I^	0.56^I^	0.45^I^	0.57^I^	0.85	-	-	-	-	-	-	-	-	-
IPQ-MH: Symptom Coherence	11.90	5.38	0–20	-0.17^**^	-0.41^I^	-0.27 ^I^	-0.28 ^I^	-0.39 ^I^	0.92	-	-	-	-	-	-	-	-	-
WHODAS 2.0: Cognition	7.20	5.46	0–24	0.32^I^	0.48^I^	0.54^I^	0.45^I^	0.49^I^	0.86	-	-	-	-	-	-	-	-	-
WHODAS 2.0: Social interaction	4.78	4.42	0–20	0.36^I^	0.54^I^	0.66^I^	0.50^I^	0.54^I^	0.82	-	-	-	-	-	-	-	-	-
WHODAS 2.0: Household	4.16	4.47	0–16	0.28^I^	0.37^I^	0.43^I^	0.35^I^	0.33^I^	0.96	-	-	-	-	-	-	-	-	-
WHODAS 2.0: Daily routine	4.27	4.24	0–16	0.30^I^	0.41^I^	0.58^I^	0.34^I^	0.46^I^	0.93	-	-	-	-	-	-	-	-	-
WHODAS 2.0: Society	7.63	6.99	0–32	0.41^I^	0.55^I^	0.77^I^	0.49^I^	0.62^I^	0.91	-	-	-	-	-	-	-	-	-
PHQ-9	-	-	-	-	-	-	-	-	-	9.54	5.98	0–24	0.33^II^	0.40^II^	0.47^II^	0.39^II^	0.48^II^	0.88
ASI-3: Cognitive	-	-	-	-	-	-	-	-	-	7.46	5.95	0–24	0.26^II^	0.42^II^	0.30^II^	0.36^II^	0.39^II^	0.88
ASI-3: Social	-	-	-	-	-	-	-	-	-	10.29	6.21	0–24	0.20^**^	0.30^II^	0.22^**^	0.19^**^	0.24^**^	0.87
ASI-3: Physical	-	-	-	-	-	-	-	-	-	7.82	5.98	0–24	0.12^†^	0.27^II^	0.20^**^	0.17^*^	0.26^II^	0.89
ASI-3: Total	-	-	-	-	-	-	-	-	-	25.57	16.06	0–72	0.22^**^	0.39^II^	0.28^II^	.27^II^	0.34^II^	0.94
DOCS: Contamination	-	-	-	-	-	-	-	-	-	2.49	3.27	0–20	0.18^**^	0.17^**^	0.13^†^	0.13^†^	0.09^†^	0.89
DOCS: Responsibility	-	-	-	-	-	-	-	-	-	3.12	3.35	0–20	0.03^†^	0.06^†^	0.07^†^	0.06^†^	0.05^†^	0.91
DOCS: Thoughts	-	-	-	-	-	-	-	-	-	3.56	3.74	0–20	0.27^II^	0.32^II^	0.41^II^	0.35^II^	0.42^II^	0.91
DOCS: Symmetry	-	-	-	-	-	-	-	-	-	2.55	3.36	0–20	0.12^†^	0.15^*^	0.20^**^	0.18^*^	0.15^*^	0.91
DOCS: Total	-	-	-	-	-	-	-	-	-	11.66	10.26	0–80	0.22^**^	0.27^II^	0.30^II^	0.27^II^	0.29^II^	0.92

*N* = 553-597 (Study 1); *N* = 178-185 (Study 2). S-Five = Selective Sound Sensitivity Syndrome Scale; EXT = *Externalising* Appraisals, INT = *Internalising* Appraisals, IMP = *Impact*, OUT = Outbursts, THR = Threat; AQ = Aggression Questionnaire; BITe = Brief Irritation Test. STICSA-T = State-Trait Inventory for Cognitive and Somatic Anxiety - Trait Scales. DERS = Difficulties in Emotion Regulation Scale; G-O Behavior = Goal-oriented behavior; Emot. Dys = Emotional Dysregulation; Gen. Dys. = General Dysregulation; NAQ = Noise Avoidance Questionnaire; BEAQ = Brief Experiential Avoidance Questionnaire. WHODAS 2.0 = World Health Organization Disability Assessment Schedule 2.0; ASI-3 = Anxiety Sensitivity Index 3; DOCS = Dimensional Obsessive Compulsive Scale; PHQ-9 = Brief Patient Health Questionnaire; McDonald’s ω based on the respective confirmatory factor analyses are in parentheses on the diagonal. M = mean, SD = standard deviation, Min = scale minimum, Max = scale maximum; *N* = 566–613 (Study 1); *N* = 178–185 (Study 2). Means were calculated for manifest sum scores of the respective scale.

a Mean and standard deviation were calculated for all DERS items.

b McDonald’s ω as defined in the bifactor S•I-1 model.

† *p* ≥ 0.05;

* *p* < 0.05;

** *p* < 0.01.

I *p* < 0.00006 (Bonferroni-corrected significance level in study 1).

II *p* < 0.0001 (Bonferroni-corrected significance level in study 2).

For both externalising and internalising appraisals, we found strong positive correlations with anger and irritation reactions in both samples (Table 4). Hostility, verbal and physical aggression, all of which are part of the definition of aggression (AQ), were found to be mainly moderately correlated with internalising and externalising appraisals, except for hostility and internalising appraisals where we found a high correlation (Table 5). Irritability (BITe) emerged to correlate highly with both appraisal styles, however, stronger with internalising appraisals (*p* < 0.0029; Table 5). This pattern could also be partly shown for the association with behavioral dysregulation (BMQ-R; Table 4) in study 1 (*p* < 0.0029), but not for study 2 (*p* = 0.013), and for outbursts in study 2 (*p* < 0.0029; Table 3), but not for study 1 (*p* = 0.014). However, for difficulties in impulse control (behavioral dysregulation; DERS) we found no correlations with misophonic appraisal styles (Table 5). We further found associations between internalising and externalising appraisals and functional impairment in different life domains: social interactions, participation in society, cognition, daily routines, and household (WHODAS 2.0; Table 5). Internalising appraisals were most strongly correlated with impairment in social interaction, society and cognition, whereas externalising appraisals were significantly lower but still moderately correlated with impairment in different life domains (*p* < 0.0029), except for impairment in household (*p* = 0.0170) and impairment in daily routine (*p* = 0.004). Further, misophonic distress and functional impairment (BMQ-R) were strongly correlated with internalising appraisals, but relatively lower with externalising appraisals in study 1 (*p* < 0.0029; Table 4), but not for study 2 (*p* = 0.054 and *p* = 0.455, respectively). We further found lower correlations between externalising appraisals and impact (S-Five) compared to internalising appraisals in study 1 (*p* < 0.001), but not for study 2 (*p* = 0.146).

Anxiety sensitivity (ASI-3) moderately correlated with internalising appraisals and; Table 3 descriptively lower with externalising appraisals (Table 6). The highest correlation was found for the cognitive facet of anxiety sensitivity with internalising appraisals, whereas the lowest (not significant) correlation emerged between the physical facet of anxiety sensitivity and external appraisals. Notably, the obsessive–compulsive facets contamination and unacceptable thoughts of the DOCS were positively weakly to moderately correlated with both misophonic appraisal styles showing no descriptive difference in the magnitude. The facets responsibility and symmetry were weakly or not correlated with misophonic appraisals. Depressive symptoms (PHQ-9) were moderately correlated with both internalising and externalising appraisals, however, not significantly lower with externalising appraisals (*p* = 0.176).

**Table 6:** Means, standard deviations and intercorrelations of the S-Five-T scores, S-Five, the BMQ-R symptom part, MisoQuest, PHQ-9, DOCS, and ASI-3.

*Measure*	TC	FIRS	RIRS	IR	DST	DIS	ANG	PAN	PHY
S-Five-T (*N* = 155)									
TC	-	0.86^II^	0.39^II^	0.16^†^	0.56^II^	0.30^II^	0.63^II^	0.11^†^	0.11^†^
FIRS	-	-	0.77^II^	−0.14^†^	0.48^II^	0.26^**^	0.71^II^	0.19^**^	0.16^*^
RIRS	-	-	-	−0.45^II^	0.22^**^	0.12^†^	0.55^II^	0.22^**^	0.14^†^
IR	-	-	-	-	−0.06^†^	−0.03^†^	−0.22^**^	−0.09^†^	−0.01^†^
DST	-	-	-	-	-	0.04^†^	0.39^II^	0.11^†^	0.04^†^
DIS	-	-	-	-	-	-	0.07^†^	0.02^†^	−0.01^†^
ANG	-	-	-	-	-	-	-	−0.02^†^	0.02^†^
PAN	-	-	-	-	-	-	-	-	0.18^*^
Min–Max	0–37	0–370	0–10	0–37	0–37	0–37	0–37	0–37	0–37
Mean (SD)	22.39 (7.67)	133.31 (68.46)	5.64 (1.91)	6.11 (4.74)	2.92 (3.41)	3.03 (2.99)	6.69 (5.28)	0.81 (1.89)	0.29 (1.26)
S-Five Factors (*N* = 155)									
Externalising	0.56^II^	0.62^II^	0.45^II^	0.07^†^	0.26^**^	0.23^**^	0.47^II^	0.11^†^	0.14^†^
Internalising	0.55^II^	0.64^II^	0.54^II^	−0.06^†^	0.34^II^	0.18^*^	0.59^II^	0.27^**^	0.12^†^
Impact	0.54^II^	0.65^II^	0.54^II^	−0.07^†^	0.36^II^	0.06^†^	0.49^II^	0.35^II^	0.12^†^
Outburst	0.52^II^	0.65^II^	0.58^II^	−0.04^†^	0.45^II^	0.16^†^	0.59^II^	0.25^**^	0.18^*^
Threat	0.55^II^	0.66^II^	0.57^II^	−0.05^†^	0.35^II^	0.05^†^	0.60^II^	0.37^II^	0.18^*^
Total	0.63^II^	0.76^II^	0.63^II^	−0.03^†^	0.41^II^	0.14^†^	0.64^II^	0.33^II^	0.14^†^
MisoQuest (*N* = 78)									
Total	0.53^II^	0.70^II^	0.69^II^	−0.21^†^	0.42^II^	0.13^†^	0.68^II^	0.29^*^	−0.04^†^
BMQ-R (*N* = 76)									
Anger	0.49^II^	0.65^II^	0.58^II^	−0.12^†^	0.32^**^	0.07^†^	0.70^II^	0.20^†^	0.16^†^
Irritation	0.43^II^	0.60^II^	0.63^II^	−0.01^†^	0.19^†^	0.08^†^	0.53^II^	0.21^†^	0.20^†^
Disgust	0.68^II^	0.77^II^	0.57^II^	−0.05^†^	0.26^*^	0.43^II^	0.70^II^	0.06^†^	0.06^†^
Physical Symptoms	0.46^II^	0.59^II^	0.57^II^	−0.09^†^	0.36^**^	0.06^†^	0.49^II^	0.29^*^	0.28^*^
Anxiety	0.37^**^	0.47^II^	0.46^II^	−0.02^†^	0.23^*^	−0.03^†^	0.44^II^	0.44^II^	0.26^*^
Behavioral Dysregulation	0.48^II^	0.61^II^	0.51^II^	−0.06^†^	0.27^*^	0.14^†^	0.64^II^	0.14^†^	0.15^†^
Recognition of Disprop.	0.37^**^	0.48^II^	0.39^**^	−0.05^†^	0.09^†^	0.20^†^	0.56^II^	0.09^†^	−0.06^†^
Recognition of Excess	0.43^II^	0.61^II^	0.59^II^	−0.12^†^	0.19^†^	0.10^†^	0.60^II^	0.26^*^	0.17^†^
Reactive Avoidance	0.46^II^	0.61^II^	0.59^II^	−0.12^†^	0.27^*^	0.12^†^	0.50^II^	0.16^†^	0.29^*^
Anticipatory Avoidance	0.39^**^	0.56^II^	0.58^II^	−0.05^†^	0.34^**^	−0.08^†^	0.33^II^	0.29^*^	0.27^*^
Distress	0.36^**^	0.53^II^	0.60^II^	−0.12^†^	0.20^†^	0.04^†^	0.43^II^	0.25^*^	0.37^**^
Functional Impairment	0.48^II^	0.62^II^	0.60^II^	−0.14^†^	0.33^**^	0.02^†^	0.42^II^	0.33^**^	0.35^**^
PHQ-9 (*N* = 145)									
Total	0.40^II^	0.45^II^	0.37^II^	−0.02^†^	0.34^II^	0.12^†^	0.26^**^	0.26^**^	0.27^**^
ASI-3 (*N* = 146)									
Cognitive	0.35^II^	0.40^II^	0.29^II^	0.11^†^	0.22^**^	0.09^†^	0.24^**^	0.25^**^	0.29^II^
Social	0.24^**^	0.23^**^	0.15^†^	0.16^†^	0.14^†^	0.11^†^	0.13^†^	0.11^†^	0.32^II^
Physical	0.22^**^	0.21^*^	0.13^†^	0.14^†^	0.22^**^	0.03^†^	0.02^†^	0.18^*^	0.20^*^
Total	0.31^II^	0.33^II^	0.24^**^	0.14^†^	0.22^**^	0.09^†^	0.16^†^	0.21^*^	0.30^II^
DOCS (*N* = 139)									
Contamination	0.23^**^	0.24^**^	0.17^*^	0.16^†^	0.04^†^	0.17^*^	0.10^†^	0.05^†^	0.17^*^
Responsibility	0.11^†^	0.08^†^	0.04^†^	0.18^*^	0.18^*^	0.02^†^	−0.05^†^	0.12^†^	0.15^†^
Thoughts	0.28^**^	0.37^II^	0.34^II^	−0.02^†^	0.20^*^	0.05^†^	0.35^**^	0.19^*^	0.02^†^
Symmetry	0.22^**^	0.20^*^	0.15^†^	0.04^†^	0.19^*^	0.12^†^	0.18^*^	0.03^†^	0.03^†^
Total	0.28^**^	0.32^II^	0.27^**^	0.08^†^	0.25^**^	0.13^†^	0.21^*^	0.16^†^	0.14^†^

*N* = 76–155; TC = total count; FIRS = frequency and intensity reaction count; RIRS = relative intensity of reactions score; RC: reaction count; IR = RC-Irritation; DST = RC-Distress; DIS = RC-Disgust; ANG = RC-Anger; PAN = RC-Panic; PHY = RC-Physiological; BMQ-R: Berlin Misophonia Questionnaire Revised; PHQ-9: Physical Health Questionnaire; ASI-3: Anxiety Sensitivity Index; DOCS: Dimensional Obsessive-Compulsive Scale. All correlations were calculated using Spearman’s correlation coefficient (ρ).

† *p* ≥ 0.05;

* *p* < 0.05;

** *p* < 0.01.

II *p* < 0.0001 (Bonferroni-corrected significance level in study 2).

#### Misophonic emotional experiences

As we did not assign any of the S-Five scales to the domain misophonic emotional experiences, we report results within this domain in a separate table (see Table 7).

**Table 7:** Latent (study 1) and Spearman’s (study 2) intercorrelations of misophonic symptoms from the domain misophonic emotional experiences.

*Measure*	1	2	3	4	5	6	7	8	9	10	11	12	13
1. BMQ-R: Anger	-	-	-	-	-	0.74^II^	-	0.77^II^	-	0.63^II^	-	-	0.73^II^
2. AQ: Anger	0.59^I^	-	-	-	-	-	-	-	-	-	-	-	-
3. AQ: Verbal Aggression	0.38^I^	0.78^I^	-	-	-	-	-	-	-	-	-	-	-
4. AQ: Physical Aggression	0.29^I^	0.54^I^	0.58^I^	-	-	-	-	-	-	-	-	-	-
5. AQ: Hostility	0.37^I^	0.70^I^	0.70^I^	0.45^I^	-	-	-	-	-	-	-	-	-
6. BMQ-R: Irritation	0.89^I^	0.50^I^	0.36^I^	0.22^**^	0.37^I^	-	-	0.68^II^	-	0.65^II^	-	-	0.68^II^
7. BITe: Irritability	0.62^I^	0.72^I^	0.48^I^	0.37^I^	0.62^I^	0.62^I^	-	-	-	-	-	-	-
8. BMQ-R: Disgust	0.57^I^	0.34^I^	0.23^I^	0.24^I^	0.26^I^	0.65^I^	0.40^I^	-	-	0.53^II^	-	-	0.61^II^
9. DPSS-R: Disgust Propensity	0.41^I^	0.39^I^	0.26^I^	0.26^I^	0.36^I^	0.40^I^	0.39^I^	0.72^I^	-	-	-	-	-
10. BMQ-R: Anxiety	0.62^I^	0.39^I^	0.29^I^	0.23^**^	0.39^I^	0.72^I^	0.40^I^	0.49^I^	0.31^I^	-	-	-	0.78^II^
11. STICSA: Cognitive Anxiety	0.55^I^	0.63^I^	0.54^I^	0.32^I^	0.85^I^	0.59^I^	0.47^I^	0.42^I^	0.46^I^	0.61^I^	-	-	-
12. STICSA: Somatic Anxiety	0.60^I^	0.53^I^	0.43^I^	0.27^I^	0.54^I^	0.63^I^	0.42^I^	0.44^I^	0.42^I^	0.73^I^	0.69^I^	-	-
13. BMQ-R: Physical Symptoms	0.81^I^	0.53^I^	0.38^I^	0.30^I^	0.42^I^	0.86^I^	0.59^I^	0.57^I^	0.40^I^	0.84^I^	0.62^I^	0.89^I^	-

*N* = 556–652 (Study 1); *N* = 102–105 (Study 2). Cells below the diagonal represent latent intercorrelations for Study 1; Cells above the diagonal represent Spearman’s correlation coefficients (*ρ*) for Study 2. The depicted correlations are rounded.

** *p* < 0.001.

I *p* < 0.00006 (Bonferroni-corrected significance level in study 1).

II *p* < 0.0001 (Bonferroni-corrected significance level in study 2).

All misophonic emotional reactions were highly correlated with physical symptoms (BMQ-R) except for verbal and physical aggression, hostility (AQ), and disgust propensity (DPSS-R), which were moderately correlated. We found high associations between anxiety and anger and irritation (BMQ-R) as well as between cognitive anxiety symptoms (STICSA) and anger, irritation (BMQ-R), and verbal aggression and hostility (AQ). Interestingly, cognitive anxiety (STICSA) was descriptively highest correlated with hostility and anger (AQ). Contrary to our prediction, anxiety was not always lower correlated with other emotional reactions than their respective intercorrelations. For example, anxiety and irritation (BMQ-R) correlated to *r* = 0.72, whereas irritation correlated lower with anger (AQ; *r* = 0.50; *p* < 0.0029) and irritability (BITe; *r* = 0.62; *p* < 0.0029). Another example is a similarly high correlation between anxiety and anger (BMQ-R) compared to the correlation between two measures of anger (BMQ-R and AQ; *r* = 0.58; *p* = 0.062). An even clearer pattern emerges for the second study, where we only found a predicted difference between the association of anxiety and disgust (*r* = 0.53) compared to disgust and anger (*r* = 0.77; *p* < 0.0029). All other correlations were not significantly different (*p* > 0.0029 for all comparisons).

#### Misophonia-specific dysregulation

Outbursts (S-Five) and threat (S-Five) were highly correlated with behavioral dysregulation (BMQ-R) in both samples. Outbursts also correlated highly with threat in both samples and further significantly higher than in the original validation study (*p* < 0.001 for both comparisons). Also, general dysregulation (DERS; BMQ-R; Tables 4 and 5) was highly correlated with outbursts and threat, whereas difficulties in impulse control (behavioral dysregulation; DERS) when controlled for general dysregulation was not significantly correlated with any S-Five measure (Table 5). As expected, emotional dysregulation (BMQ-R) was highly correlated with threat, but emotional dysregulation when controlled for general dysregulation (DERS) was not significantly associated with threat. Notably, we also found a strong association between cognitive dysregulation (BMQ-R) and threat.

Besides correlations within the domain misophonia-specific dysregulation, threat was very strongly associated with misophonic anxiety (BMQ-R) as well as cognitive and somatic anxiety symptoms (STICSA). Likewise, physical misophonic symptoms were strongly associated with threat. In line with predictions about associations with avoidance behavior, we found high correlations between threat and reactive and anticipatory avoidance (BMQ-R), noise avoidance (NAQ), and behavioral avoidance (BEAQ).

Symptoms within the domain misophonic impairment were predominantly strongly associated with threat. For example, impact (S-Five) as well as distress and functional impact (BMQ-R) correlated strongly with threat in both samples (Table 4). Also, specifically impairment in society and social interaction (WHODAS 2.0) were strongly associated (Table 5), whereas impairment in cognition, daily routine and household were moderately correlated. Likewise, strong associations between outbursts and impact (S-Five), functional impact and distress (BMQ-R), as well as impairments in social interaction (WHODAS 2.0) were observed. Moderate associations emerged for outbursts and impairment in household, daily routine, and society (WHODAS 2.0).

Depressive symptoms (PHQ-9) were moderately associated with both threat and outbursts (Table 6). For anxiety sensitivity (ASI-3), we found low to moderate associations with threat and outbursts, descriptively being slightly higher for threat than for outbursts. Obsessive–compulsive thoughts (DOCS) were moderately associated with threat and outbursts, whereasother OCD symptoms were not significantly correlated.

Low to moderate correlations emerged with anxiety sensitivity (ASI-3), depressive symptoms (PHQ-9), and some obsessive–compulsive traits: unacceptable thoughts and symmetry (DOCS), but not with contamination and responsibility (Table 6).

#### Misophonic avoidance

All avoidance symptoms were highly correlated with threat and impact (S-Five). For example, reactive avoidance and anticipatory avoidance (BMQ-R) were highly correlated with threat and impact in both samples (Table 4). Furthermore, noise avoidance (NAQ) and behavioral avoidance (BEAQ) were highly correlated with threat and impact. Descriptively, these correlations were higher than any other correlation between avoidance symptoms and other S-Five scales.

#### Misophonic impairment

Most predicted associations between symptoms from the domain misophonic impairment and other misophonic symptoms have already been described in the preceding sections. Simply summarized, all S-Five scales were expected to be strongly associated with misophonic impairment symptoms. We found high correlations for all S-Five scales with functional impact and distress (BMQ-R; Table 4), except for a moderate correlation between distress and externalising appraisals in the second study. Further, impairments in different life domains (WHODAS 2.0) were moderately to strongly associated with all S-Five scales. However, impairments in household (compared to other life domains) emerged to correlate descriptively lower with all S-Five scales on average (see Table 5).

Impact was further moderately correlated with depressive symptoms (PHQ-9), cognitive symptoms of anxiety sensitivity (ASI-3) and obsessive–compulsive thoughts (DOCS; see Table 6). Low correlations were observed for social and physical symptoms of anxiety sensitivity and obsessive–compulsive symmetry symptoms. Other obsessive–compulsive symptoms were not significantly correlated with impact.

#### Associations with symptoms of other mental disorders and traits.

The correlations of the five reaction counts (irritation, distress, disgust, anger, panic, physiological response) and the three S-Five-T indices (TC, FIRS, RIRS) with the S-Five factors, the BMQ-R, ASI-3 and DOCS scores are presented in Table 6.

The number of triggers (TC) selected from the 37 sounds list was strongly correlated with all S-Five dimensions (*r* > 0.50 in all cases). Correlations of similar magnitude emerged with the MisoQuest total score, the BMQ-R scales of misophonic anger, irritation, disgust, physical reactions, behavioral dysregulation, reactive avoidance, and functional impairment. The strongest correlation emerged with misophonic disgust reactions and, unexpectedly, the lowest with distress (both BMQ-R). However, depressive symptoms (PHQ-9) correlated moderately high. Low or non-significant coefficients emerged with anxiety sensitivity (ASI-3) and obsessive–compulsive traits (DOCS).

For the FIRS index very strong correlations with the total S-Five, the total MisoQuest and the BMQ-R disgust reaction scale (*r* > 0.70) emerged. Further, all S-Five factors were highly correlated with the FIRS index. Moderate to moderate high were also the correlations between FIRS and the rest of the BMQ-R subscales. Further, we observed moderately high correlations with depressive symptoms (PHQ-9). As in the case of the TC, low or non-significant coefficients emerged for most anxiety sensitivity (ASI-3), and most obsessive–compulsive traits (DOCS). However, moderate correlations were observed for cognitive anxiety sensitivity and unacceptable thoughts. Similar patterns emerged for the RIRS index, even though coefficients were descriptively somewhat smaller in all cases.

With respect to the reaction counts (RC), RC-Irritation did not show significant correlations with any of the scales.

RC-Distress was moderately to moderately low associated with most scales. The strongest correlations appeared between distress and the S-Five outburst factor and the total MisoQuest. Distress was not found to be significantly correlated with all BMQ-R scales, with social anxiety sensitivity (ASI-3) and the DOCS scales. Interestingly, externalising appraisals was the only S-Five subscale which was not significantly correlated with RC-distress.

RC-Disgust also did not relate with most of the subscales considered. An exception was the moderate correlation with disgust reaction (BMQ-R).

On the contrary, RC-Anger was strongly related to all S-Five scores, MisoQuest, and all BMQ-R scores, with a lower correlation only with anticipatory avoidance. Non-significant coefficients emerged between RC-Anger and depressive symptoms (PHQ-9), anxiety sensitivity (ASI-3), and obsessive–compulsive symptoms (DOCS).

Interestingly, RC-Panic did not correlate with the S-Five externalising and internalising appraisals and outburst factor but correlated moderately with threat and impact. RC-Panic also correlated moderately with the BMQ-R anxiety reaction scale while moderately low correlations emerged with the BMQ-R scales of physical reactions, recognition of excess, anticipatory avoidance, distress, and functional impairment, however, these associations were not significant when considering Bonferroni-correction. RC-Panic did not correlate with the other BMQ-R scales. Low but non-significant correlations emerged between RC-Panic and PHQ-9, all ASI-3 factors apart from the social factor, and with the DOCS thought factor.

Finally, the RC-Physiological did not correlate with the S-Five factors or the total MisoQuest. Moderate correlations emerged with the BMQ-R scales of physical and anxiety reactions, reactive and anticipatory avoidance, distress, and functional impairment, however, these associations were not significant when considering Bonferroni-correction. Low but non-significant correlations emerged between the RC-Physiological and the total PHQ-9, and the contamination scale of the DOCS. The only significant associations were found between RC-Physiological and the ASI-3 total as well as the ASI-3 cognitive and social subscale.

#### Associations with overall misophonic symptoms (S-Five).

Overall misophonic symptoms (MisoQuest) strongly correlated with each of the S-Five subscales with externalising appraisals being lowest correlated and threat being highest correlated (see Table 4). The scales were further strongly associated with general sound intolerance symptoms (BMQ-R; *r* > 0.60).

## Discussion

The presented studies aimed at providing a rigorous and valid German translation of the S-Five. We thus presented a thorough examination of the reliability and construct validity by specifying measurement models and introducing a nomological network which delineates associations between misophonic symptoms.

Our results demonstrate a good fit of the five-factor model to our data in both samples when using the translated S-Five items, emerging a similar fit to the English version (*cf.*, Vitoratou et al., 2021b). However, some misspecifications were identified which need further investigation in future studies. We therefore presented a promising, alternative model, which incorporates a bifactor S-1 measurement model for misophonic outbursts. This has three advantages: the model (a) fits better to the data (even when penalizing for more parameters), (b) provides a clearer interpretation of different aspects of outbursts, and (c) preserves a general outburst factor with a clearer interpretation. Besides the goodness of fit of the factorial structure, we investigated measurement invariance regarding gender and age. Based on findings of minor effects, we conclude that the German S-Five items do not function differentially due to gender and age, and therefore structural differences of the scores can be assessed.

We also demonstrated excellent internal consistency in both samples and high test–retest reliability for the five factors. As an interim conclusion, these results reveal two main properties of the German S-Five: a) highly reliable measurement and b) factorial valid conclusions when applying these scales. Another striking result is the mostly replicated correlation pattern between S-Five factors with medium to strong intercorrelations. Unexpectedly, the factors were in general higher correlated than in the original validation study. Interestingly, threat is highly correlated with each of the four remaining factors, especially with impact. Further, threat was comparably higher correlated with internalising appraisals and outbursts than other factors. We argue that these are beneficial properties of the threat scale, however, users of the S-Five should keep the small differences in the correlative pattern in mind when administering the German version. Initial evidence on the construct validity was shown through high correlations with measures of overall misophonic symptoms, however, this does not allow to disentangle which misophonic symptoms are correlated with the S-Five scales. Therefore, we developed the nomological network of misophonic symptoms.

### Construct validity and the nomological network

To our knowledge this is the first study that begins to explore a formal and comprehensive nomological network for misophonic symptoms. Recent developments of misophonia instruments, which emphasize a more symptom-oriented measurement (Rosenthal et al., 2021; Remmert et al., 2022) and the German translation of the S-Five provide the basis to scrutinize the proposed nomological network with five broader symptom domains, in which the symptoms are proposed to be clustered. These domains are misophonic appraisals, misophonic emotional experiences, misophonia-specific dysregulation, misophonic avoidance, and misophonic impairment. Our aims were to explicitly provide evidence for the construct validity of the (translated) S-Five as well as giving a deeper insight into the associations of misophonic symptoms.

We found strong evidence for the construct validity of internalising and externalising appraisals in the misophonic appraisals domain. Internalising appraisals were strongly associated with the recognition of the excessive and disproportionate nature of the reactions and furthermore higher correlated with these dimensions of clinical insight than externalising appraisals. This aligns with similar findings from other mental disorders (e.g., Cotton et al., 2012; Didehbani et al., 2012).

Problem coherence, that is, having a good understanding of your problem, was negatively correlated with internalising appraisals, as expected. We had also expected the relative difference between internalising and externalising appraisals in their relationship with coherence (with externalising appraisals not being correlated) but had not anticipated these negative correlations across all factors. These relationships were in the opposite direction to those between the S-Five and variables about awareness of the disproportionate and excessive nature of reactions, indicating that awareness of these aspects is not the same as having a good understanding of the problem. So, what we labelled as “insight” on the nomological network is a reflection of awareness that misophonic symptoms are indeed a problem, but not necessarily insight *into* the problem. This finding was consistent with research finding that OCD severity is negatively associated with problem coherence (Pedley et al., 2019). Further research is needed to test possible explanations for this. Perhaps the simplest understanding of this relationship is that misophonia is easier to make sense of when it is less severe. It is also important to consider that there could be a causal relationship in the other direction. That is, it is possible that as one’s understanding of the problem of misophonia improves, their symptoms decrease. Lack of an explanation for the problem may be, in fact, part of the problem. Our cross-sectional correlative study does not allow us to draw any conclusions about causality. Future research would therefore benefit from testing problem coherence as a potential mechanism of change for misophonia. This would make sense for internalising in particular, which is characterized by a felt sense that the individual with misophonia is reacting this way to sounds because of some deeper character flaw, being a bad or angry person underneath. It thus makes sense that as one comes to understand a theory that misophonia is a decreased sensory tolerance problem shared by many and shaped by our experiences, that their previous theory of “bad character” loses its credibility.

Besides associations within the domain misophonic appraisal, we also found evidence for construct validity through associations between internalising and externalising appraisals with symptoms from other domains. For example, we found that both appraisal styles were at least moderately associated to anger, aggression, irritability, and behavioral dysregulation and outbursts, which is in line with previous findings on these appraisal styles (Vitoratou et al., 2021b). Considering general psychological theories of appraisals and their associations with anger and related constructs (e.g., Averill, 1983) it is a rather contradicting result, but there seems to be a difference for misophonia, which has been replicated in our studies. Further studies should consider investigating the role of appraisals for experiencing misophonic anger, aggression, and potential outbursts. An unpredicted result was the non-significant correlation between difficulties in impulse control (behavioral dysregulation) and appraisals, which does not support our hypotheses on appraisals. Note that we also found mixed results for comparing associations of externalising and internalising appraisals with outbursts and behavioral dysregulation, which do neither support equally high associations nor higher associations with either appraisal style. Further research should investigate these relationships in depth.

Lastly, we observed medium to strong associations with different symptoms from the domain misophonic impairment, which were almost all higher for internalising appraisals than for externalising appraisals. Only the association between the S-Five scale impact was equally high for internalising and externalising appraisals in our second study, which contradicted our hypotheses on the associations with impact. Furthermore, we found depressive symptoms to be equally moderately correlated with both appraisal styles. Thus, our results show evidence that both appraisal styles might be associated with impact on lives of affected individuals as well as with respective depressive symptoms. We strongly suggest investigating how both appraisal styles are associated with misophonic impairment exploring possible explanatory variables.

Within the domain of misophonic emotional experiences we found strong evidence for our hypothesis that physical symptoms of misophonia are strongly associated with all emotional symptoms, which again replicates the results from past studies (e.g., Rosenthal et al., 2021). While we found strong associations between explicitly convergent measures of the same emotional reaction, we found strong evidence against our hypothesis that anxiety is differently related to other misophonic emotional reactions. Especially in the second study almost all associations between anger, irritation, disgust and anxiety were as high as the associations among anger, irritation and disgust, respectively. Mind that due to the small sample size in study 2, interpretations should be made cautiously. However, the findings of study 1 also support the conclusion that anxiety is not weaker associated with all other emotional reactions. Although Jager et al. (2020) see anxiety reactions as a subordinate misophonic symptom, others have pointed out that anxiety is a crucial symptom (e.g., Swedo et al., 2022). Our findings give further evidence that anxiety is strongly related to other emotional reactions and hence a paramount emotional symptom to be considered when investigating misophonia.

For the validation of the S-Five the domain misophonia-specific dysregulation plays a particularly important role since two scales were assigned to this domain: outbursts and perceived threat. As expected, outbursts and threat were strongly associated not only among each other but with different facets of misophonia-specific dysregulation. Outbursts were predominantly strongly related to convergent measures of behavioral dysregulation and threat was strongly related to emotional dysregulation. We further found strong associations with threat and anxiety and physical symptoms, which replicate findings from the original validation study (Vitoratou et al., 2021b). Both threat and outbursts were expectedly strongly associated with symptoms from misophonic impairment, especially with functional impact, distress, and impairment in social interaction.

Interestingly, we found an exploratory association of threat with symptom coherence. Regarding the possible impact of lack of coherence on an increased sense of emotional threat, which includes feelings of being trapped, helpless and distress, it also makes sense that one might experience a greater sense of these in a moment where their initial reactions do not make sense to them, thus compounding the overall reaction. This theory would provide support for the S-Five concept of threat to fall within the domain of dysregulation rather than affectivity. Our assumption was that threat would show stronger associations with anxiety. However, not only anxiety but other emotional reaction correlated strongly with threat, too. Additionally, threat was strongly correlated with emotional and cognitive dysregulation, and moderately associated with general and behavioral dysregulation and both types of avoidance. While the term “threat” may denote a sense of fear, these results indicate that it is not an anxiety response, but rather a more complex emotional and cognitive experience. We propose that the sense of emotional threat comes from a combination of the initial emotional reaction, compounded by a lack of understanding of the problem, and a sense of not being able to cope (dysregulation) in that moment, resulting in a feeling of being trapped, panicked and helpless if unable to get away from the situation. This could be explored further in qualitative studies seeking to understand the complexity of what is happening in these moments.

As expected, there were also strong associations for threat with avoidance. Future experimental research would be helpful to determine whether avoidance plays a maintaining role in the sense of threat experienced by those with misophonia. Further, symptoms of avoidance were strongly associated with the S-Five scale impact. Future studies could investigate how avoidance and coping strategies are related to impairment.

The domain misophonic impairment has been shown to be a crucial domain insofar that symptoms from this domain, and especially impact as measured by the S-Five, were highly correlated with symptoms from all other domains. Further, all scales from the S-Five were highly associated with impact. Although impact plays an important role in the nomological network because it is related to a wide range of misophonic symptoms, future studies should investigate the causes of impact. This study provides a basis to select variables that have been shown to be strongly associated. With regard to the S-Five-T reactions, we found that misophonia severity was strongly associated with the number of times anger was reported as a primary reaction to triggers, supporting the frequent reporting of anger as the predominant response in misophonia (Brout et al., 2018; Jager et al., 2020), at least with regard to the primary reaction to trigger sounds. Reports of panic as a primary reaction were also associated with misophonia severity, which is supported by the findings of Vitoratou et al. (2021b), but is contrary to the suggestion by Jager et al. (2020) that anxiety and panic should not be considered a primary reaction in misophonia.

It was interesting to note that the count of physiological reaction was not associated with overall misophonia severity but was associated with anxiety sensitivity. One possible explanation for this is that the physiological reaction reported in misophonia (Edelstein et al., 2013; Kumar et al., 2017) may be mostly a physical manifestation of emotions. While there may be some individuals who experience only a physiological reaction, it’s also possible that some report it as physiological if they are not able to identify or label specific emotions, especially if they show high traits of anxiety sensitivity, which measure fear of anxiety symptoms. Further in-depth investigation is needed to understand this better.

We found that the reaction count of irritation as a primary reaction to sounds was not associated with misophonia severity, nor any of the related scales. However, the S-Five factors were all positively associated with the BMQ-R measure of irritation and irritability more generally. This supports the notion proposed by Vitoratou et al. (2021b), that while irritation may be part of the experience of misophonia, if someone reports that irritation is their most frequent response to trigger sounds, that is likely not indicative of the disorder of misophonia, and in fact represents a typical response to unpleasant sounds reported in the general population (Vitoratou et al., 2022a). Finally, we looked at the S-Five in relation to symptoms of depression and obsessive–compulsive disorder. In line with previous studies, misophonia severity was associated with symptoms of depression (Wu et al., 2014; Erfanian et al., 2019; Jager et al., 2020), particularly with regards to threat and impact (Vitoratou et al., 2021b). Threat was moderately associated with anxiety sensitivity, which warrants further investigation to expand on previous work examining misophonia and anxiety sensitivity (Cusack et al., 2018; McKay et al., 2018; Schadegg et al., 2021). In line with previous research (Cusack et al., 2018; McKay et al., 2018), symptoms of misophonia were associated with some aspects of OCD symptoms but not others, with moderate correlations with unacceptable thoughts and low correlations with symmetry. This adds to growing evidence that misophonia is not specifically part of obsessive–compulsive and related disorders (McKay et al., 2018). Further research could investigate overlapping transdiagnostic mechanisms, for example intrusive thoughts and urges in misophonia and potential related beliefs around the likelihood of acting on those intrusions.

Overall, the results have shown that symptoms measured by the S-Five fit well in the proposed nomological network of misophonic symptoms, which provides strong evidence for the construct validity of the (German) S-Five. The studies have also replicated past results from studies with the S-Five and revealed unknown exploratory associations of the S-Five scales with misophonic symptoms and symptoms of other mental disorders.

We hope that the proposed nomological network is understood as a first attempt to formalize further investigations of misophonic symptoms and thus provide a structural and theoretical basis. Furthermore, this article aims to raise awareness of a symptom-oriented approach to investigate misophonia and thus help readers and future research to understand associations between misophonic symptoms and how to disentangle and explain them.

## Limitations

Although two large and independent samples were drawn, we did not implement a random sampling scheme. Our samples were drawn from social media which is why our results are not representative for the German population (e.g., more women, more highly educated, younger individuals were sampled) and are therefore biased and difficult to generalize. However, as we aimed at gathering data mainly from affected individuals, there is no alternative random sampling strategy applicable. We suggest administering the German S-Five in a large representative sample to assess the psychometric properties for the German population without sampling bias. Also note that for some of the analyses in study 2, the sample sizes were rather small (*N* = 76–155), which should be considered in the interpretation of results. We hence strongly recommend interpreting results, which stem from these smaller samples, with caution and replicating them.

The surveys lasted more than 40 min on average which might have caused exhaustion and higher dropout rates, but we implemented a rigorous data quality assessment which certainly minimized this issue. Nevertheless, we cannot guarantee unbiased estimates due to systematic missing responses or exhaustion.

A methodological issue limiting the scope of our results is the exclusive administration of questionnaires. Podsakoff et al. (2003) demonstrated artificially increasing correlations due to shared method-specific variance. We therefore suggest an extension of the study using different measurement methods (e.g., interviews and behavioral data). Another limitation of our study is that we did not assess hyperacusis (i.e., a decreased sound tolerance condition related to misophonia, which is mainly characterized by aversive reactions to physical characteristics of sounds such as loudness; e.g., Jastreboff and Jastreboff, 2014) as a measure of discriminant validity which should be addressed in follow-up studies.

Since misophonia is still a relatively little investigated condition, we did not fulfill strong properties of a nomological network. Thus, a major weakness of our study is the network was based on observations from the misophonia and broader literature, rather than being derived from a comprehensive theoretical framework, which does not yet exist for the etiology and maintenance of misophonia. Our observed associations therefore need to be further corroborated. The proposed nomological network should be interpreted as a first attempt to formalize and disentangle associations between misophonic symptoms. This attempt is thus deemed to stimulate further development of a more rigorous and extended nomological network in future research. A more profound nomological network for misophonia is dependent on substantiated theories on misophonic processes, requiring theoretical models with testable hypotheses. Our study provided a formalized and reasonable first approach to a nomological network for misophonia, one which will need to be further tested and refined.

## Conclusion

In summary, the presented nomological network overall clearly supports the validity of the German S-Five and gives comprehensive insight into the relationship of misophonic symptoms in general. The demonstrated measures to capture symptoms of misophonia have been shown to be psychometrically robust and allow for reliable and valid conclusions.

## Data availability statement

The original contributions presented in the study are publicly available. This data can be found here: https://osf.io/qswyt/files/osfstorage.

## Ethics statement

The studies involving human participants were reviewed and approved by Ethics Committee at the Department of Education and Psychology of the Freie Universität Berlin and PNM Research Ethics Panel, King’s College London. The patients/participants provided their written informed consent to participate in this study.

## Author contributions

NR planned, conducted and analyzed the first study, provided supervision to the second study, participated in the translation, and contributed to the manuscript. AJ participated in the translation, collected the data and performed part of the analysis for the second study, and contributed to the manuscript. RG participated in the translation and reviewed the manuscript. JG contributed to the introduction and discussion sections of the manuscript. SV provided supervision to both studies, completed the analysis, and contributed to the manuscript. All authors contributed to the article and approved the submitted version.

## Funding

SV was funded by the Biomedical Research Centre for Mental Health at South London and Maudsley NHS Foundation Trust and King’s College London. This research was funded in whole, or in part, by the Wellcome Trust (JG; Grant number 102176/B/13/Z).

## Conflict of interest

The authors declare that the research was conducted in the absence of any commercial or financial relationships that could be construed as a potential conflict of interest.

## Publisher’s note

All claims expressed in this article are solely those of the authors and do not necessarily represent those of their affiliated organizations, or those of the publisher, the editors and the reviewers. Any product that may be evaluated in this article, or claim that may be made by its manufacturer, is not guaranteed or endorsed by the publisher.

## Author disclaimer

The views expressed are those of the author(s) and not necessarily those of the NHS, The Wellcome Trust, the NIHR or the Department of Health and Social Care.

## Footnotes

^1^Translated and reproduced with permission from the test authors.

^2^Reproduced under the terms of the Creative Commons Attribution License (CC BY-NC 4.0).

^3^Translated and reproduced with permission from the test authors.

^4^Reproduced with permission from the test authors.

^5^Reproduced with permission from the test authors.

^6^Reproduced under the terms of the Creative Commons Attribution License (CC BY-NC-SA 3.0 IGO).

^7^Reproduced with permission from the test authors.

^8^Reproduced under the terms of the Creative Commons Attribution License (CC BY-NC-SA 3.0 IGO).

^9^Reproduced with permission from the test authors.

^10^Fulfillment of the diagnostic criteria was determined by proving if each criterion is fulfilled. A criterion was considered as fulfilled if the mean of the respective symptom scale was greater than 3 (i.e., greater than the mean of the response scale, meaning self-reported approval of the items). For severe symptoms, the fulfillment cutoff was set to 4. Note that this scheme was not empirically derived, but chosen pragmatically because there is no empirical criterion to determine the fulfillment of the criteria.
