# Supplementary material for: A nomological network for misophonia in two German samples using the S-Five model for misophonia
Source: Front Psychol. 2022 Dec 21;13:902807. doi: 10.3389/fpsyg.2022.902807 (PMC9811822; doi:10.3389/fpsyg.2022.902807)
Supplement: Supplementary file 1 [file Data_Sheet_1.docx]

Supplementary Material

# Supplementary Figures and Tables

## Table 1. The S-Five in English, with scoring information

| A. The S-Five Statements |
| --- |
| Please read each statement carefully and base your answer on how true they feel to you based on your current thoughts, experiences, and reactions*: 0-not at all true to 10-completely true* |
| *Externalising* |
| EXT01 People should not make certain sounds, even if they do not know about others' sensitivities. |
| EXT02 I get angry at other people because of how disrespectful they are with the noises they make. |
| EXT03 People should do everything they can to avoid making noises that might bother others. |
| EXT04 I react strongly to certain sounds because I cannot stand how selfish, thoughtless, or bad-mannered people can be. |
| EXT05 Certain sounds are just bad manners, and it is not strange to feel intense anger about that. |
| *Internalising* |
| INT01 The way I react to certain sounds makes me wonder whether deep inside I am just a bad person. |
| INT02 The way I react to certain noises makes me feel like I must be an unlikable person deep down. |
| INT03 I respect myself less because of my responses to certain sounds. |
| INT04 I feel like I must be a very angry person inside because of the way I react to certain sounds. |
| INT05 I dislike myself in the moments of my reactions to sounds. |
| *Impact* |
| IMP01 My job opportunities are limited because of my reaction to certain noises. |
| IMP02 I do not meet friends as often as I would like to because of the noises they make. |
| IMP03 There are places I would like to go but do not, because I am too worried about how the noises will impact me. |
| IMP04 I can see future where I cannot do everyday things because of my reactions to noises. |
| IMP05 The way I feel/react to certain sounds will eventually isolate me and prevent me from doing everyday things. |
| *Outburst* |
| OUT01 I can get so angry at certain noises that I get physically aggressive towards people to make them stop. |
| OUT02 Sometimes I get so distressed by noises that I use violence to try and make it stop. |
| OUT03 Some sounds are so unbearable that I will shout at people to make them stop. |
| OUT04 If people make certain sounds that I cannot bear, I become verbally aggressive. |
| OUT05 I am afraid I will do something aggressive or violent because I cannot stand the noise someone is making. |
| *Threat* |
| THR01 I feel trapped if I cannot get away from certain noises. |
| THR02 I feel anxious if I cannot avoid listening to certain sounds. |
| THR03 If I cannot get away from certain noises, I am afraid I might panic or feel like I will explode. |
| THR04 If I cannot avoid certain sounds, I feel helpless. |
| THR05 I can experience distress as the result of some noises. |

**Scoring:**

In this section each item is rated on a 11-point rating scale, for example:

People should not make certain sounds, even if they do not know about others’ sensitivities

0 (not at all true) 1 2 3 4 5 6 7 8 9 10 (completely true)

**Factor scores and Total Score:** Please add the responses of the corresponding items for each factor to compute the factor score and all items for the total S-Five-E score. Each factor has 5 items thus the scores are directly comparable in terms of statement endorsement.

**Range:** Factor scores range between 0 and 50, total score is between 0 and 250.

| B. The S-Five-T Trigger Checklist |
| --- |
| **Trigger reaction items:** Thinking about the past few weeks, what is the main feeling this sound* has caused you? *No feeling, irritation, distress, disgust, anger, panic, other feeling: negative, other feeling: positive, other: physiological reaction* |
|  |
| **Trigger intensity items:** Thinking about the past few weeks, please rate the intensity of your reaction to this sound* when made by another person or object *(from 0: doesn’t bother me at all, to, 10: unbearable/causes suffering)* |
|  |
| *List of triggers currently included in the S-Five-t: Normal eating sounds, Certain letter sounds, Mushy foods being eaten, Sound of clipping nails, Swallowing, Keyboard tapping, Lip smacking, Normal breathing, Repetitive engine noises, Loud/unusual breathing, Mobile phone sounds, Repetitive coughing, Humming noise, Repetitive sniffing, Snoring, Certain accents, Whistling sound, Sound of tapping, Rustling, Chewing gum, Footsteps, Hiccups, Slurping, Cutlery noises, Sneezing, Certain words, Kissing, Joint cracking, Muffled sounds, Throat clearing, Baby crying, Repetitive barking, Loud chewing, Clock ticking, Crunching eating sounds, Teeth sucking, Yawning. |

The S-Five-T is made in a flexible format to allow researchers and treatment providers to customise the checklist according to the needs of their study/client. That is, its format facilitates adding or removing triggers as research findings progress or when treatment plans are being customised. More importantly, the format of the items allows to add or remove reactions.

Here we use 37 triggers and 9 reactions (no feeling to psychological reaction). We derive four useful summary indices from the S-Five-T checklist, according to the definitions and scoring guidelines described in Vitoratou et al. (2021b).

**Scoring:**

**Trigger Count (TC)**: the total number of triggers endorsed by a participant from the list provided. So, for the present version, the participant’s TC would take values between 0-37, as we listed 37 possible triggers. For example, if an individual selected ‘no feeling’ or ‘other: positive’ reaction to 32 out of 37 triggers, their TC would be 5, that is, the number of triggers to which they experience a negative reaction (irrespective of intensity). This index tells us about the number of triggers that cause a negative reaction.

**Reaction Count (RC)**: is the number of times each reaction type is endorsed and can be counted across triggers in a single participant, or across participants. The index is computed for each reaction type separately, resulting in a reaction count for each (i.e. RC-Anger, RC-Irritability, etc). The total possible RC for a participant is determined by the number of triggers listed (i.e. 37 in the present study). For example, if a participant selected anger as their main emotional reaction to three triggers, and panic as their main reaction to two triggers, and no feeling for their remaining triggers, then they would have an RC-Anger of 3, RC-Panic of 2 and RC-No feeling of 32 (irrespective of intensity). This index tells us about the nature of the emotional responses to triggers.

**Frequency/Intensity of Reactions Score (FIRS)**: the total value of the intensity items of all endorsed triggers. The intensity is rated from 0-10, and therefore for the present study, the FIRS takes values between 0 and 370. For example, if a participant reported a negative reaction to 5 triggers and rated each to the highest possible intensity (that is, 10), their FIRS value would be 50. A participant who reported 10 triggers, each at a moderate intensity of 5, would also have a FIRS value of 50. This index provides combined information about the number of triggers and their intensity.

**Relative Intensity of Reactions Score (RIRS)**: gives an estimate of the intensity of reactions to triggers, relative to the number of triggers reported. It is computed by dividing the FIRS index by the TC index. RIRS takes values between 0 and 100, regardless of the number of reactions available and number of triggers listed in the study. Continuing with the examples from above, the individual who had a FIRS of 50, who reported 5 triggers with an intensity of 10 each, their RIRS would equal 10 (50 divided by 5 triggers). But the individual with the same FIRS (50) who reported 10 triggers with an intensity of 5, would have a RIRS of 5 (FIRS 50 divided by TC 10). This index provides information about the average intensity of an individual’s reaction to triggers.

The scoring guide and the programming codes (SPSS, R, Stata) to obtain all factors and indices are freely available upon request made to Silia Vitoratou ([silia.vitoratou@kcl.ac.uk](mailto:silia.vitoratou@kcl.ac.uk))

## Table 2. The S-Five in German, with scoring information

*Translated by: Nico Remmert, Rebecca Gruzman, Antonia Jebens*

| 1. Das S-Five |
| --- |
| Bitte lesen Sie jede Aussage sorgfältig durch und stützen Sie Ihre Antwort darauf, wie wahr sie Ihnen auf der Grundlage Ihrer derzeitigen Gedanken, Erfahrungen und Reaktionen erscheint: *0 = überhaupt nicht zutreffend bis 10 = vollkommen zutreffend* |
| *Externale Bewertung* |
| EXT01 Menschen sollten bestimmte Geräusche nicht machen, selbst wenn sie nicht über die Empfindlichkeiten anderer Bescheid wissen. |
| EXT02 Ich ärgere mich über andere Menschen, weil sie so respektlos Geräusche machen. |
| EXT03 Man sollte alles daran setzen, keine Geräusche zu machen, die andere vielleicht stören könnten. |
| EXT04 Ich reagiere stark auf bestimmte Geräusche, weil ich es nicht ausstehen kann, wie egoistisch, unbedacht und unhöflich Leute sein können. |
| EXT05 Bestimmte Geräusche sind einfach unhöflich und es ist nicht verwunderlich, deshalb stark verärgert zu sein. |
| *Internale Bewertung* |
| INT01 So wie ich auf bestimmte Geräusche reagiere, gibt mir zu denken, ob ich tief in mir drin einfach eine schlechte Person bin. |
| INT02 So wie ich auf bestimmte Geräusche reagiere, gibt mir das Gefühl, tief in mir drin ein unsympathischer Mensch sein zu müssen. |
| INT03 Ich respektiere mich weniger wegen meiner Reaktionen auf bestimmte Geräusche. |
| INT04 Ich habe das Gefühl, dass ich tief in mir drin eine wütende Person bin, aufgrund der Art und Weise wie ich auf bestimmte Geräusche reagiere. |
| INT05 In den Momenten, in denen ich auf Geräusche reagiere, mag ich mich nicht. |
| *Auswirkungen* |
| IMP01 Meine Berufsmöglichkeiten sind aufgrund meiner Reaktion auf bestimmte Geräusche begrenzt. |
| IMP02 Ich treffe mich nicht so oft mit Freunden, wie ich es gerne würde, wegen der Geräusche, die sie machen. |
| IMP03 Es gibt Orte, die ich gerne besuchen würde, jedoch gehe ich nicht hin, weil ich mich zu sehr darüber sorge, wie sich die Geräusche auf mich auswirken werden. |
| IMP04 Ich kann mir eine Zukunft vorstellen, in der ich wegen meiner Reaktionen auf Geräusche keine alltäglichen Dinge mehr tun kann. |
| IMP05 Die Art und Weise, wie ich mich fühle/auf bestimmte Geräusche reagiere, wird mich letztendlich isolieren und mich daran hindern, alltägliche Dinge zu tun. |
| *Ausbrüche* |
| OUT01 Ich kann mich über bestimmte Geräusche so sehr ärgern, dass ich körperlich aggressiv gegenüber Menschen werde, um sie zum Aufhören zu bringen. |
| OUT02 Manchmal bringen mich bestimmte Geräusche so dermaßen in Aufruhr, dass ich gewalttätig werde, um es zu stoppen. |
| OUT03 Manche Geräusche sind so unerträglich, dass ich Menschen anschreie, damit sie aufhören. |
| OUT04 Wenn Menschen bestimmte Geräusche machen, die ich nicht aushalten kann, dann werde ich verbal aggressiv. |
| OUT05 Ich habe Angst, dass ich etwas Aggressives oder Gewalttätiges tun werde, weil ich ein Geräusch nicht aushalte, das jemand macht. |
| *Bedrohung* |
| THR01 Ich fühle mich gefangen, wenn ich bestimmten Geräuschen nicht entkommen kann. |
| THR02 Ich fühle mich beunruhigt, wenn ich es nicht vermeiden kann, bestimmte Geräusche zu hören. |
| THR03 Wenn ich bestimmten Geräuschen nicht entkommen kann, fürchte ich, dass ich vielleicht in Panik ausbreche oder es sich so anfühlt, als würde ich jeden Moment explodieren. |
| THR04 Wenn ich bestimmte Geräusche nicht vermeiden kann, dann fühle ich mich hilflos. |
| THR05 Es kann vorkommen, dass ich ein Gefühl von Not erlebe, wenn ich manche Geräusche höre. |

**Operationalisierung**

**Instruktionen**: Sie werden gleich einige Fragen in Bezug auf Geräuschempfindlichkeiten beantworten. Bitte lesen Sie jede Aussage sorgfältig durch und stützen Sie Ihre Antwort darauf, wie wahr sie Ihnen auf der Grundlage Ihrer derzeitigen Gedanken, Erfahrungen und Reaktionen erscheint.

**Antwortformat**:

11-stufige Likert-Skala (0 = überhaupt nicht zutreffend bis 10 = vollkommen zutreffend)

| 1. Die S-Five-T Trigger Checkliste |
| --- |
| **Trigger Reaktion:** Wenn Sie an die letzten paar Wochen zurückdenken, was ist das hauptsächliche Gefühl, das dieses Geräusch* bei Ihnen ausgelöst hat?  *Kein Gefühl, Irritation, Aufgewühlt fühlen, Ekel, Wut, Panik, andere Gefühle: negative, andere Gefühle: positive, andere: physiologische Reaktionen* |
|  |
| **Trigger Intensität:** Wenn Sie über die letzten paar Wochen nachdenken, bewerten Sie bitte die Intensität Ihrer Reaktion auf dieses Geräusch*, ausgelöst durch eine andere Person oder einen Gegenstand. (v*on 0: stört mich gar nicht, bis 10: nicht auszuhalten/verursacht Leiden)* |
|  |
| * Liste der Triggergeräusche, die derzeit in der Trigger Checkliste enthalten sind: Normale Essgeräusche, bestimmte Laute, das Kauen von weichlichem Essen, das Geräusch von Nägel schneiden, Schlucken, Tastaturtippen, Schmatzen, normales Atmen, sich wiederholende Motorengeräusche, lautes/ungewöhnliches Atmen, Handygeräusche, sich wiederholendes Husten, Brummen, sich wiederholendes Schniefen, Schnarchen, bestimmte Akzente, Pfeifgeräusche, leise Klopfgeräusche, Geraschel, Kaugummikauen, Schrittgeräusche, Schluckauf, Schlürfen, Geräusche durch Besteck, Niesen, bestimmte Wörter, Kussgeräusche, Knacken von Gelenken, gedämpfte Geräusche, Räuspern, das Weinen von Babys, wiederholtes Hundegebell, lautes Kauen, das Ticken einer Uhr, knuspernde Essgeräusche, Zähne ansaugen, Gähnen. |

**Auswertung:**

**Triggerzählung (TZ):** Die Gesamtzahl der Trigger, die von den Teilnehmenden aus der vorgegebenen Liste bestätigt wurden. In der vorliegenden Version würde die TZ des/der Teilnehmers/-in also Werte zwischen 0 und 37 annehmen, da wir 37 mögliche Trigger aufgelistet haben. Wenn eine Person beispielsweise "kein Gefühl" oder "andere: positive" Reaktion auf 32 von 37 Triggern auswählt, würde die TZ 5 betragen, d. h. die Anzahl der Trigger, auf die sie eine negative Reaktion (unabhängig von der Intensität) erfährt. Dieser Index gibt Aufschluss über die Anzahl der Trigger, die eine negative Reaktion hervorrufen.

**Reaktionszählung (RZ):** Die Häufigkeit, mit der jeder Reaktionstyp bestätigt wird. Dies kann für alle Trigger einer einzelnen Person oder für alle Teilnehmenden gezählt werden. Der Index wird für jeden Reaktionstyp separat berechnet, was zu einer Reaktionszahl für jeden Typ führt (d.h. RZ-Wut, RZ-Irritation etc.). Die mögliche Gesamt-RZ einer Person wird durch die Anzahl der aufgeführten Trigger bestimmt (in der vorliegenden Studie 37). Wenn ein/eine Teilnehmer/-in beispielsweise Wut als die hauptsächliche emotionale Reaktion auf drei Trigger angibt, Panik als die hauptsächliche Reaktion auf zwei Trigger, und für die übrigen Trigger kein Gefühl angibt, dann hätte die Person ein RZ-Wut von 3, ein RZ-Panik von 2 und ein RZ-Kein Gefühl von 32 (unabhängig von der Intensität). Dieser Index gibt Aufschluss über die Art der emotionalen Reaktionen auf Trigger Geräusche.

**Häufigkeit/Intensität des Reaktionswertes (HIR):** Der Index ist der Gesamtwert der Trigger Intensitäten aller Trigger. Die Trigger Intensität wird auf einer Skala von 0 bis 10 bewertet, weshalb der HIR in der vorliegenden Studie Werte zwischen 0 und 370 annimmt. Wenn Teilnehmende beispielsweise eine negative Reaktion auf 5 Trigger angibt und jeden mit der höchstmöglichen Intensität (d. h. 10) bewertet, würde der HIR-Wert 50 betragen. Eine Person, die 10 Auslöser mit einer mittleren Intensität von jeweils 5 meldete, hätte ebenfalls einen HIR-Wert von 50. Dieser Index liefert kombinierte Informationen über die Anzahl der Auslöser und deren Intensität.

**Relative Intensität des Reaktionswertes (RIR):** ein Schätzwert für die Intensität der Reaktionen auf Trigger im Verhältnis zur Zahl der gemeldeten Trigger. Er wird berechnet, indem der HIR-Index durch den TZ-Index geteilt wird. RIR nimmt Werte zwischen 0 und 100 an, unabhängig von der Anzahl der verfügbaren Reaktionen und der Anzahl der in der Studie aufgeführten Trigger. Um bei den obigen Beispielen zu bleiben: Die Person mit einem HIR von 50, welche 5 Trigger mit einer Intensität von jeweils 10 angegeben hat, hätte einen RIR-Index von 10 (50 geteilt durch 5 Trigger). Die Person mit dem gleichen HIR-Wert (50), welche 10 Trigger mit einer Intensität von 5 meldete, hätte dagegen eine RIR-Index von 5 (HIR 50 geteilt durch TZ 10). Dieser Index gibt Auskunft über die durchschnittliche Intensität der Reaktion einer Person auf Trigger.
